# Different projection neurons of basolateral amygdala participate in the retrieval of morphine withdrawal memory with diverse molecular pathways

**DOI:** 10.1038/s41380-023-02371-x

**Published:** 2023-12-26

**Authors:** Xinli Guo, Yu Yuan, Xiaoman Su, Zixuan Cao, Chenshan Chu, Chao Lei, Yingqi Wang, Li Yang, Yan Pan, Huan Sheng, Dongyang Cui, Da Shao, Hao Yang, Yali Fu, Yaxian Wen, Zhangyin Cai, Bin Lai, Ming Chen, Ping Zheng

**Affiliations:** 1 State Key Laboratory of Medical Neurobiology, Institutes of Brain Science, MOE Frontier Center for Brain Science, Department of Neurology of Zhongshan Hospital, Fudan University, Shanghai, 200032 China; 2 Medical College of China Three Gorges University, Yichang, 443002 China

**Keywords:** Neuroscience, Molecular biology

## Abstract

Context-induced retrieval of drug withdrawal memory is one of the important reasons for drug relapses. Previous studies have shown that different projection neurons in different brain regions or in the same brain region such as the basolateral amygdala (BLA) participate in context-induced retrieval of drug withdrawal memory. However, whether these different projection neurons participate in the retrieval of drug withdrawal memory with same or different molecular pathways remains a topic for research. The present results showed that (1) BLA neurons projecting to the prelimbic cortex (BLA^-PrL^) and BLA neurons projecting to the nucleus accumbens (BLA^-NAc^) participated in context-induced retrieval of morphine withdrawal memory; (2) there was an increase in the expression of Arc and pERK in BLA^-NAc^ neurons, but not in BLA^-PrL^ neurons during context-induced retrieval of morphine withdrawal memory; (3) pERK was the upstream molecule of Arc, whereas D1 receptor was the upstream molecule of pERK in BLA^-NAc^ neurons during context-induced retrieval of morphine withdrawal memory; (4) D1 receptors also strengthened AMPA receptors, but not NMDA receptors, -mediated glutamatergic input to BLA^-NAc^ neurons via pERK during context-induced retrieval of morphine withdrawal memory. These results suggest that different projection neurons of the BLA participate in the retrieval of morphine withdrawal memory with diverse molecular pathways.

## Introduction

Drug addiction is a chronic, relapsing brain disorder characterized by compulsive drug seeking [1]. A typical framework for drug addiction includes a three-stage cycle: binge/intoxication, withdrawal/negative affect, and preoccupation/anticipation [2]. The binge/intoxication stage mainly involves the rewarding effects of drugs of abuse and development of incentive salience [2, 3]; the withdrawal/negative affect stage mainly involves the increases in negative emotional states and dysphoric and stress-like responses [2, 4]; the preoccupation/anticipation stage involves the craving and deficits in executive function [2]. During the three-stage cycle, memory surrounding drug use/ withdrawal is formed and becomes exceptionally strong. This addiction memory, irreversibly imprinted in the brain, can be retrieved when persons with substance dependence disorders encounter environmental context that is previously associated with drug rewarding or drug withdrawal symptoms [5]. The retrieval of both opioid rewarding memory or opioid withdrawal memory is important driver of opioid relapse. Therefore, the successful treatment of addictive disorders requires an approach that addresses neural basis underlying context-induced retrieval of drug addiction memory. It would not only help in developing new therapeutic approaches to prevent drug relapse, but also be of important significance for understanding neural basis of the retrieval of memory.

Previous studies have studied neural basis underlying context-induced retrieval of morphine withdrawal memory. Conditioned place or food aversion (CPA or CFA) is typical animal model, in which aversive response to morphine withdrawal-paired compartment is used as the index of context-induced retrieval of morphine withdrawal memory [6, 7]. Using CPA or CFA model, previous studies have examined the brain regions that contribute to context-induced retrieval of morphine withdrawal memory, such as the basolateral amygdala (BLA) [8], the hippocampus [9], the paraventricular nucleus of the thalamus (PVT) [10], the nucleus accumbens (NAc) [11]. In addition, at neural circuit level, it has been reported that context can activate prelimbic cortex (PrL) neurons projecting to the BLA (PrL^-BLA^ neurons) [12, 13], PVT neurons projecting to the NAc (PVT^-NAc^ neurons) [14], NAc neurons projecting to the LH (NAc^-LH^ neurons) [15, 16] and the inhibition of PrL^-BLA^ or PVT^-NAc^ or NAc^-LH^ neurons can significantly inhibit context-induced retrieval of morphine withdrawal memory, suggesting that these projection neurons participate in context-induced retrieval of morphine withdrawal memory. However, whether these different projection neurons participate in the retrieval of morphine withdrawal memory in diverse molecular pathways remains a topic for further study.

To investigate this topic, firstly, we studied whether context could activate BLA^-PrL^ and BLA^-NAc^ neurons in morphine withdrawal mice by examining c-Fos expression using immunofluorescence staining method, and the role of these neurons in context-induced retrieval of morphine withdrawal memory by examining the influence of the inhibition of these neurons, using chemogenetic method, on context-induced retrieval of morphine withdrawal memory. Second, we studied whether context induced a neural plasticity change in BLA^-PrL^ neurons or BLA^-NAc^ neurons by examining the change in the expression of Arc, a marker of neuronal plasticity [17], using immunofluorescence staining, fluorescence-activated cell sorting (FACS) and qRT-PCR methods, during context-induced retrieval of morphine withdrawal memory in morphine withdrawal mice. Third, we studied the upstream molecular pathways that induced an increase in the expression of Arc after exposure to context in BLA^-NAc^ neurons in morphine withdrawal mice. At last, using electrophysiological recording technique, we studied the strengthening effect of D1 receptor on AMPA receptor-mediated glutamatergic input to BLA^-NAc^ neurons during context-induced retrieval of morphine withdrawal memory.

## Materials and methods

### Experimental animals

Male adult (8–12 weeks) C57BL/6 J mice were housed under 12 h light/dark cycle in a temperature (23 ± 1 °C)- and humidity (40%)-controlled environments with free access to food and water. All experimental procedures conformed to Fudan University as well as international guidelines on the ethical use of animals, and were approved by the Animal Care and Use Committee of the Shanghai Medical College of Fudan University (No. 20200306-148). All efforts were made to minimize animal suffering and reduce the number of animals used.

### Surgery

Mice were anesthetized by intraperitoneal (IP) injection of sterile tribromoethanol (20 mg/ml, 300 mg/kg) before the surgery, and secured in a stereotaxic instrument (Stoelting). Microinjections were performed using glass electrode connected to a 1 μl microsyringe (Hamilton) by polyethylene tubing and controlled by a syringe pump (Harvard Apparatus).

For retrograde labeling experiments, mice received bilateral injections of FluoroGold (FG, 0.3 μl, 4% dissolved in PBS; Fluorochrome, USA) into the NAc (anteroposterior (AP), +1.64 mm; mediolateral (ML), ±1.22 mm; dorsoventral (DV), −4.57 mm) or the PrL (AP, +1.98 mm; ML, ±0.30 mm; DV, −2.20 mm).

For in vivo chemogenetic (DREADD) inhibition in conditioned place aversion (CPA) experiments, mice were injected with the AAV2/9 rAAV-EF1a-DIO-hM4D(Gi)-Enhanced Green Fluorescent Protein (EGFP)-WPREs (AAV-DIO-hM4Di-EGFP) virus or the same viral vectors carrying GFP alone (AAV-DIO-EGFP, 5.71×10^12^ vector genomes/ml; Brain VTA, China) bilaterally into the BLA (AP, −1.4 mm; ML, ± 3.4 mm; DV, −4.90 mm) at a volume of 0.3 μl, and AAV2/2Retro-hsyn-CRE-mCherry-WPRE-PA (AAV-retro-cre-mCherry, 5.0 × 10^12^ vector genomes/ml; Brain VTA, China) into the NAc or the PrL at a volume of 0.3 μl.

For ERK1 or ERK2 knockdown, mice were injected with the pAAV-CMV-DIO-EGFP-MIR30shRNA (ERK1) virus (AAV-DIO-MIR30-shERK1) or pAAV-CMV-bGlobin-Flex-EGFP-MIR30shRNA (ERK2) virus (AAV-Flex-MIR30-shERK2) or the same viral vectors carrying EGFP alone (4.0 × 10^12^ vector genomes/ml; OBIO Technology, China) bilaterally into the BLA at a volume of 0.3 μl, and AAV2/2Retro-hsyn-CRE-mCherry-WPRE-PA (AAV-retro-cre-mCherry, 5.0 × 10^12^ vector genomes/ml; Brain VTA, China) into the NAc at a volume of 0.3 μl. After the virus injection, the mice were returned to the home cages for recovery and virus expression for 2–3 weeks before behavioral assay.

For local infusion of SCH23390 (0.5 μg/side) or Sulpiride (1 μg/side), stainless-steel guide cannula was bilaterally embedded 1 mm above the BLA. The cannula was immobilized on the skull with anchoring screws and dental cement. To prevent occlusion, stainless-steel stylets were inserted into the cannula. After the surgery, mice were allowed to recover for a week before the behavioral assay.

### Chronic morphine treatment

Mice were treated with morphine (Shenyang No.1 Pharmaceutical Factory, China), as described before [12]. Briefly, morphine dependence was induced in mice by repeated intraperitoneal injections of morphine twice daily at 09.00 AM and 19.00 PM. Morphine doses were progressively increased from 10 mg/kg to 40 mg/kg: day 1, 2 × 10 mg/kg; day 2, 2 × 20 mg/kg; day 3, 2 × 30 mg/kg; day 4 and 5, 2 × 40 mg/kg. Control mice were treated with saline following the same procedure.

### Conditioned place aversion (CPA)

**Fig. 1:**
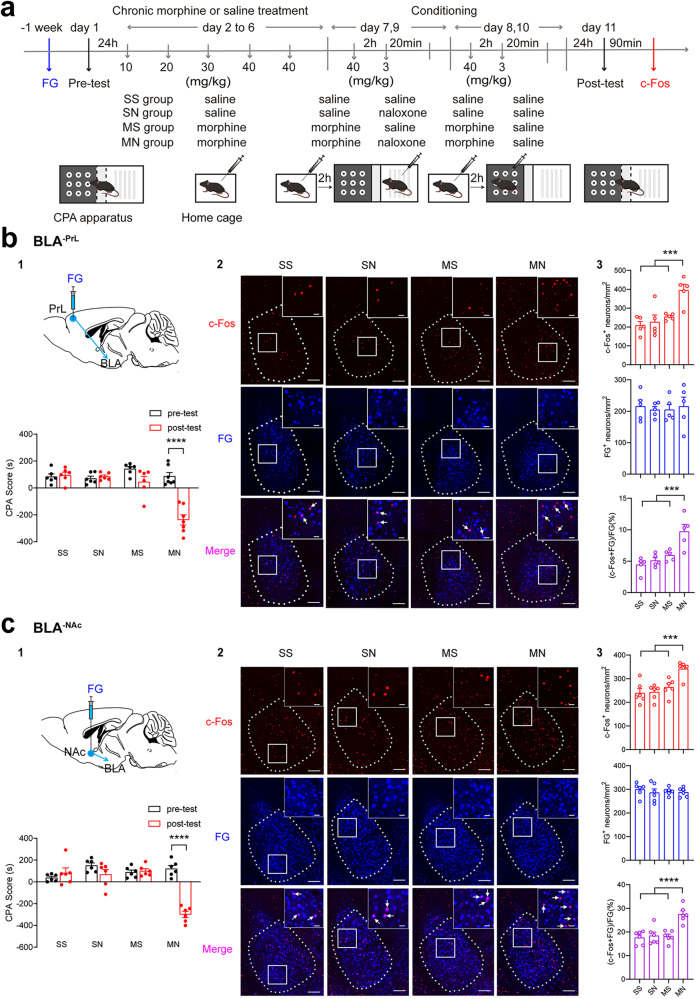
Effect of conditioned context on the expression of c-Fos in BLA^-PrL^ or BLA^-NAc^ neurons during context-induced retrieval of morphine withdrawal memory. **a** Experimental timeline for CPA procedure. **b** Effect of conditioned context on the expression of c-Fos in BLA^-PrL^ neurons during the retrieval of morphine withdrawal memory. **b1** Top: diagram of the injection of fluorogold (FG) into the PrL. Bottom: average CPA scores in SS (*n* = 6), SN (*n* = 6), MS (*n* = 6), and MN (*n* = 7) groups. Two-way ANOVA, **** *P* < 0.0001. **b2** Top: c-Fos-positive neurons in the BLA. Center: FG-labeling neurons in the BLA. Bottom: co-labeling neurons of c-Fos and FG in the BLA. Scale bar, 100 μm. Magnified image shows the boxed area. Scale bar, 20 μm. **b3** Top: quantification of c-Fos-positive neurons/mm^2^ in the BLA (*n* = 5 mice in each group). One-way ANOVA, *** *P* < 0.001. Center: quantification of FG-positive neurons/mm^2^ in the BLA. One-way ANOVA, *P* > 0.05. Bottom: quantification of co-labeling ratio of c-Fos and FG relative to FG in BLA^-PrL^ neurons. One-way ANOVA, ***P < 0.001. **c** Effect of conditioned context on the expression of c-Fos in BLA^-NAc^ neurons during the retrieval of morphine withdrawal memory. **c1** Top: diagram of the injection of FG into the NAc. Bottom: average CPA scores in SS (*n* = 6), SN (*n* = 6), MS (*n* = 6), and MN (*n* = 6) groups. Two-way ANOVA, ****P < 0.0001. **c2** Top: c-Fos-positive neurons in the BLA. Center: FG-labeling neurons in the BLA. Bottom: co-labeling neurons of c-Fos and FG in the BLA. Scale bar, 100 μm. Magnified image shows the boxed area. Scale bar, 20 μm. **c3** Top: quantification of c-Fos-positive neurons/mm^2^ in the BLA (*n* = 6 mice in each group). One-way ANOVA, ***P < 0.001. Center, quantification of FG-positive neurons/mm^2^ in the BLA. One-way ANOVA, *P* > 0.05. Bottom, quantification of co-labeling ratio of c-Fos and FG relative to FG in BLA^-NAc^ neurons. One-way ANOVA, ****P < 0.0001. Means ± SEMs.

CPA was conducted using a three-compartment place conditioning apparatus (Med Associates, USA) with distinct visual and tactile context, and the procedure was similar to that described previously [18, 19]. As shown in the top panel of Fig. 1B, the CPA procedure included four phases: pre-test (day 1), morphine dependence (days 2–6), conditioning (days 7–10), and post-test (day 11). Firstly, mice were given a pre-test and allowed to freely explore the entire apparatus for 15 min. Mice with a strong unconditioned preference (>75% of the session time) for any compartment were discarded from the study. All mice with biased preferences were randomly divided into four groups: saline + saline (SS), saline + naloxone (SN), morphine + saline (MS), morphine + naloxone (MN). Morphine dependence was induced in mice by repeated intraperitoneal injections of morphine twice daily at 9:00 AM and 19:00 PM for 5 days, as described above. On days 7 and 9, 2 h after 40 mg/kg morphine administration, mice in the MN group were confined in morphine withdrawal-paired compartment (minor preference compartment during pre-test) for 20 min immediately after intraperitoneally injection of naloxone (3 mg/kg). On alternating days 8 and 10, 2 h after 40 mg/kg morphine administration, mice in the MN group were confined in saline-paired compartment (opposite compartment) for 20 min immediately after intraperitoneally injection of saline. The post-test was conducted 24 h after conditioning on day 10 and mice were allowed to freely explore the three compartments for 15 min. CPA score was calculated as the time spent in the naloxone-precipitated withdrawal-paired chamber minus the time spent in the no withdrawal-paired chamber.

### Immunohistochemistry and imaging

Mice were anesthetized by IP injection of sterile tribromoethanol (20 mg/ml, 300 mg/kg) and transcardially perfused with 0.9% saline, followed by ice-cold solution of 4% paraformaldehyde (PFA) in phosphate-buffered saline (PBS) (pH 7.4). The brains were removed and fixed in 4% PFA at 4 °C overnight. Then brains were sectioned into 40-μm-thick coronal slices using a vibratome (Leica, USA) and collected in PBS. For immunohistochemistry experiments, brain slices were first washed in PBS (3 × 10 min), then blocked with 10% normal goat serum and 0.3% Triton X-100 (PBS) at 37 °C for 2 h and then incubated with primary antibody at 4 °C overnight. After rinsing in PBS (3 × 10 min), the slices were incubated with biotinylated secondary antibody at 37 °C for 1 h. Then they were rinsed in PBS (3 × 10 min) and incubated with Streptavidin−Cy3™ (1:1000; S6402, Sigma, USA) or Alexa Fluor® 488 Streptavidin (1:1000, 405235, biolagend, USA) at 37 °C for 1 h. Finally, slices were cover-slipped on anti-quenching mounting medium (Thermo Fisher Scientific). All primary antibodies were dissolved into 10% normal goat serum and 0.3% Triton X-100 in PBS, and other antibodies were dissolved into 10% normal goat serum in PBS. For primary antibodies, we used antibodies against c-Fos (rabbit, 1:1000; 226003, Synaptic Systems, Germany), Arc (rabbit, 1:1000; 156003, Synaptic Systems, Germany), pERK (rabbit, 1:200; 4370, Cell Signaling Technology, USA), ERK2 (rabbit, 1:300; ab32081, abcam, USA) and CaMKIIα (rabbit, 1:1,000; ab52476, Abcam, USA). For the secondary antibody, we used biotinylated goat anti-rabbit antibody (1:500; BA-1000, Vector, USA). Images were obtained by confocal microscopy with a 20× immersion lens and collected at a resolution of 1024 × 1024 pixels. The same laser and scanning settings were used for all confocal images within an experiment to allow for comparison across groups. In general, coronal sections from 5 to 7 mice were used for quantitative analysis. Counts collected from at least 5 slices from each mouse were averaged to produce a value. Quantification of labeled neurons was performed with ImageJ software with the same threshold. The positive cells were defined with staining above basal background. All counting experiments were conducted blinded to the experimental group.

### Fluorescence-activated cell sorting

FG-injected mice were used to purify projection neurons by fluorescence-activated cell sorting (FACS). Mice were anesthetized by IP injection of sterile tribromoethanol (20 mg/ml, 300 mg/kg) and brains were extracted and placed in cold Hank’s Balanced Salt Solution (HBSS;21-022-CV, CORNING) without calcium chloride and magnesium chloride. The BLA was carefully separated from the brain and tissues from three mice were finely minced with a scalpel prior to digestion. Chopped brain tissues were placed in MACS C tubes (130-093-237, Miltenyi Biotec, Germany) with tissue dissociation enzyme (Neural Tissue Dissociation Kit, 130-093-231, Miltenyi Biotec, Germany) and processed with a mechanic dissociator (GentleMACS Octo Dissociator, 130-095-937, Miltenyi Biotec, Germany). The brain tissue homogenates were then filtered through a 70 μm cell strainer (BS-70-XBS, biosharp). Percoll™ (GE Healthcare, 17-0891-02) gradients were used to separate neurons from brain tissue homogenate. A stock solution of isotonic Percoll (SIP) was prepared (9:1 Percoll in 10× HBSS, 14185, Gibco). Cell pellets were resuspended in a 30% (v/v) and 30–70% (v/v) SIP (in 1x HBSS). For the 30% Percoll gradient, 3 ml of SIP were added to 7 ml of 1× HBSS containing the cell suspension in a 15 ml tube. The 30–70% Percoll gradient was performed by adding 3 ml of 70% SIP diluted with 1x HBSS to the bottom of 15 ml tube containing 30% SIP with the cell suspension. The tubes were centrifuged at 800 rcf for 30 min at 18 °C without acceleration or deceleration. After centrifugation, cells were collected from the middle layer (4 ml interphase) in the 30–70% Percoll gradient. Collected cells were washed by 1× HBSS and centrifuged at 800 rcf for 10 min at 18 °C and then resuspended in 1 mL HBSS with 2% BSA. Flow cytometry were performed on FACSCalibur cytometer (moflo xpp, Beckman Coulter, USA) using CellQuest Pro software (Becton Dickinson, USA).

### Quantitative real-time PCR

Specific target reverse transcription and amplification was performed using Single-Cell Sequence-Specific Amplification Kit (P621-01, Vazyme, China) following the manufacturer’s instructions. Firstly, various sets of primers were mixed together to prepare an Assay Pool (primer final concentration 0.1 µM). Reverse transcription and pre-amplification were performed in 5 μl of RT-PreAmp Master Mix. Subsequently, the sample was incubated at 50 °C for 60 min and then at 95 °C for 3 min, followed by 17 cycles of 95 °C for 15 s and 60 °C for 15 min to complete the first round of amplification. The pre-amplified complementary DNA (cDNA) was diluted 1:5 with nuclease-free ddH_2_O and stored at −20 °C until used. Quantitative RT-PCR was performed on the mastercycler ep realplex (Eppendorf) using the Hieff qPCR SYBR Green Master Mix (Yeasen, China) according to the manufacturer’s instructions (95 °C for 5 min followed by 40 cycles at 95 °C for 10 s, 60 °C for 20 s, and 72 °C for 20 s.). Primers for specific amplification were synthesized by Synbio. The relative expression value of the target gene was calculated as the ratio of target cDNA to Gapdh. Data analysis was carried out using the 2^−ΔΔCt^ method.

The following primers were used for PCR amplification:

**Table:** 

Arc-F	GTTAGCCCCTATGCCATCACC
Arc-R	CTGGCCCATTCATGTGGTTCT
D1-F	ATGGCTCCTAACACTTCTACCA
D1-R	GGGTATTCCCTAAGAGAGTGGAC
ERK2-F	CAGGTGTTCGACGTAGGGC
ERK2-R	TCTGGTGCTCAAAAGGACTGA
ERK1-F	TCCGCCATGAGAATGTTATAGGC
ERK1-R	GGTGGTGTTGATAAGCAGATTGG
GAPDH-F	TGGCCTTCCGTGTTCCTAC
GAPDH-R	GAGTTGCTGTTGAAGTCGCA

### Homogeneous time-resolved fluorescence (HTRF^®^)-based pERK assay

The HTRF^®^ Phospho-ERK kit (64ERKPEG, Cisbio Bioassays, USA) was used to test the expression of pERK. 12 μl of projection neurons sorted by FACS were transferred to 96-well cell culture plate. Projection neurons were then lysed with supplemented lysis buffer and incubate for at least 30 min at a room temperature under shaking. Then, 16 μl of lysate was made up to the final volume of 20 μl /well with 2 μl of anti-ERK1/2-Europium/Terbium Cryptate and 2 μl of anti-Phospho-ERK1/2-d2 antibody solutions were prepared in the detection buffer. The plate was then incubated for 4 h at room temperature before reading the fluorescence emission at 620 and 665 nm using a Tecan SPARK plate reader (Tecan Group Ltd.).

### Electrophysiological recording

Mice were anesthetized by IP injection of sterile tribromoethanol (20 mg/ml, 300 mg/kg) and transcardially perfused with 20 ml of ice-cold and oxygenated (95% O_2_, 5% CO_2_) cutting solution containing the following (in mM): 93 N-Methyl-D-glucamine diatrizoate (NMDG), 2.5 KCl, 10 MgSO_4_ 7H_2_O, 1.2 NaH_2_PO_4_ 2H_2_O, 30 NaHCO_3_, 25 Glucose, 10 HEPES, 5 Na ascorbate, 3 Na pyruvate, 2 Thiourea, 0.5 CaCl_2_ (280–300 mOsm, pH 7.3–7.4). The brain was rapidly removed from the skull and placed in ice-cold and oxygenated cutting solution. Serial coronal slices (250 μm) containing the BLA were prepared using a vibratome (VT-1200, Leica, Germany) and were allowed to equilibrate for 45 min at 32 °C in incubation solution containing (in mM): 92 NaCl, 2.5 KCl, 2 MgSO_4_ 7H_2_O, 1.2 NaH_2_PO_4_ 2H_2_O, 30 NaHCO_3_, 25 Glucose, 20 HEPES, 3 Na pyruvate, 2 Thiourea, 2 CaCl_2_ (280–300 mOsm, pH 7.3–7.4). Slices were transferred one at a time to the recording chamber and perfused with 32 °C oxygenated recording artificial cerebrospinal fluid (ACSF) containing (in mM): 126 NaCl, 2.5 KCl, 1.3 MgSO_4_ 7H_2_O, 1.0 NaH_2_PO_4_ 2H_2_O, 26.2 NaHCO_3_, 11 Glucose, 2.5 CaCl_2_ (280–300 mOsm, pH 7.3–7.4) at a rate of 1.5 ml min^−1^. Slices were prepared for whole-cell voltage-clamp recording using a computer-controlled amplifier (MultiClamp200B, Molecular Devices; USA). The traces were low-pass filtered at 3 kHz and digitized at 10 kHz (DigiData 1440 A, Molecular Devices). BLA^-NAc^ neurons labeled with fluorescent microspheres (488/560 EX/EM) were visualized using infrared differential interference contrast and fluorescent microscopy. Electrodes had a resistance of 3–4 MΩ when filled with the internal pipette solution.

AMPA miniature excitatory synaptic currents (mEPSCs) were recorded at a holding potential of −70 mV in the presence of tetrodotoxin (TTX) (1 μM), gabazine (10 μM) and D-AP5 (50 μM) to block the Na^+^ channels, GABA_A_ and NMDA receptors, respectively. The postsynaptic AMPA current were recorded by puffing AMPA (10 μM) directly to the labeled BLA^-NAc^ neurons at a holding potential of −70 mV in the presence of TTX (1 μM), gabazine (10 μM), D-AP5 (50 μM). The postsynaptic NMDAR current were recorded by puffing NMDA (100 μM) directly to the labeled BLA^-NAc^ neuron at a holding potential of −40 mV in the presence of TTX (1 μM), gabazine (10 μM), CNQX (10 μM). The distance was 150 μm between the tip of the puff pipette and the recorded neuron. Pressure application was controlled by a pneumatic picopump (PV820, World Precision Instruments, USA), with an inter-pulse interval of at least 1 min. The internal pipette solution contained (in mM):140 K-Gluconate, 0.1 CaCl_2_, 2 MgCl_2_, 1 EGTA, 2 K2-ATP, 0.1 Na_3_-ATP and 10 HEPES (280–300 mOsm, pH 7.3–7.4). The data-recording segments under SKF38393 and U0126 + SKF38393 were regarded as valid only after superfusing SKF38393 or U0126 and SKF38393 for at least 5 min. We replaced MgCl_2_ with CaCl_2_ in the extracellular solution to minimize the Mg^2+^ block of NMDARs at negative holding potentials.

### Quantification and statistical analyses

All experimental data were analyzed using GraphPad Prism 8 and shown as Means ± SEMs. Statistical significance was determined using Student’s *t* test for comparisons between two groups or analyses of variance (ANOVAs) for comparisons among three or more groups. One-way ANOVA was followed by Tukey’s multiple comparisons test and two-way ANOVA was followed by Bonferroni multiple comparisons test to calculate p values (treatment with different drugs as the between-subject factors and test condition as the within-subjects factor). The mEPSCs were detected and analyzed using MiniAnalysis (Synaptosoft). Puffing AMPA- and NMDA-EPSC was analyzed using Clampfit (Axon Instruments). For all results, *P* < 0.05 was considered statistically significant.

## Results

### BLA^-PrL^ and BLA^-NAc^ neurons participate in context-induced retrieval of morphine withdrawal memory

Projection neurons of the BLA are thought to be glutamatergic neurons [20, 21]. To confirm the type of BLA^-PrL^ neurons and BLA^-NAc^ neurons, we injected FluoroGold (FG) into the PrL or the NAc to retrograde label BLA^-PrL^ or BLA^-NAc^ neurons in mice, respectively. Then, we examined whether these projection neurons were glutamatergic neurons, using CaMKIIα as the marker of glutamatergic neurons [21], by immunofluorescence staining method. The results showed that FG (blue color) was co-labeled (cyan color) with CaMKIIα (green color) in most neurons of BLA^-PrL^ (93%, *n* = 6 mice; Supplementary Fig. S1a) or BLA^-NAc^ (93%, *n* = 5 mice; Supplementary Fig. S1b). These results suggest that both BLA^-PrL^ and BLA^-NAc^ neurons are glutamatergic neurons.

To study the role of BLA^-PrL^ or BLA^-NAc^ neurons in context-induced retrieval of morphine withdrawal memory, we examined whether context activated BLA^-PrL^ or BLA^-NAc^ neurons using c-Fos as a marker of neuronal activaton [22] and the influence of the inhibition of BLA^-PrL^ or BLA^-NAc^ neurons on context-induced retrieval of morphine withdrawal memory in morphine withdrawal mice. We injected FG into the PrL to retrograde label BLA^-PrL^ neurons. FG-injected mice were divided into four groups: the saline + saline (SS) group, in which the saline-treated mice were trained to CPA with saline; the saline + naloxone (SN) group, in which the saline-treated mice were trained to CPA with naloxone; the morphine + saline (MS) group, in which the chronic morphine-treated mice were trained to CPA with saline; the morphine + naloxone (MN) group, in which the chronic morphine-treated mice were trained to CPA with naloxone. One week after recovery from the surgery of FG injection, mice in each group experienced CPA paradigm (Fig. 1a). The results showed that the mice in the MN group exhibited a strong aversion to the withdrawal-paired compartment and spent less time in it during the post-test, resulting in an increase in CPA score compared with that of pre-test, whereas mice in other groups did not exhibit a significant aversion to either compartment (Two-way ANOVA, drug treatment factor, F _(3, 21)_ = 16.97, *P* < 0.0001; test condition factor, F _(1, 21)_ = 32.40, *P* < 0.0001; interaction factor, F _(3, 21)_ = 22.48, *P* < 0.0001. Bottom panel of Fig. 1b1,). The animals were sacrificed at ninety minutes after post-test and the expression of c-Fos in BLA^-PrL^ neurons was examined. The result showed that the co-labeling of c-Fos and FG in BLA^-PrL^ neurons was significantly increased in the MN group compared with that of the SS group, the SN group and the MS group (One-way ANOVA, F _(3, 16)_ = 11.66, *P* < 0.001. Bottom panel of Fig. 1b3). We also injected FG into the NAc to retrograde label BLA^-NAc^ neurons and examined the expression of c-Fos of BLA^-NAc^ neurons after post-test of CPA by the same method used in the above study of BLA^-PrL^ neurons. The results showed that the mice in the MN group exhibited a strong aversion to the withdrawal-paired compartment and spent less time in it during the post-test, resulting in an increase in CPA score compared with that of pre-test, whereas mice in other groups did not exhibit a significant aversion to either compartment (Two-way ANOVA, drug treatment factor, F _(3, 20)_ = 15.39, *P* < 0.0001; test condition factor, F _(1, 20)_ = 33.01, *P* < 0.0001; interaction factor, F _(3, 20)_ = 29.95, *P* < 0.0001. Bottom panel of Fig. 1c1). After the post-test, the animals were sacrificed at 90 min and the expression of c-Fos in BLA^-NAc^ neurons was examined. As shown in Fig. 1c1, the co-labeling of c-Fos and FG in BLA^-NAc^ neurons was significantly increased in the MN group compared with that of the SS group, the SN group and the MS group (One-way ANOVA, F _(3, 20)_ = 13.31, *P* < 0.0001. Bottom panel of Fig. 1c3). These results suggest that context can activate BLA^-PrL^ and BLA^-NAc^ neurons during the retrieval of morphine withdrawal memory in morphine withdrawal mice.

**Fig. 2:**
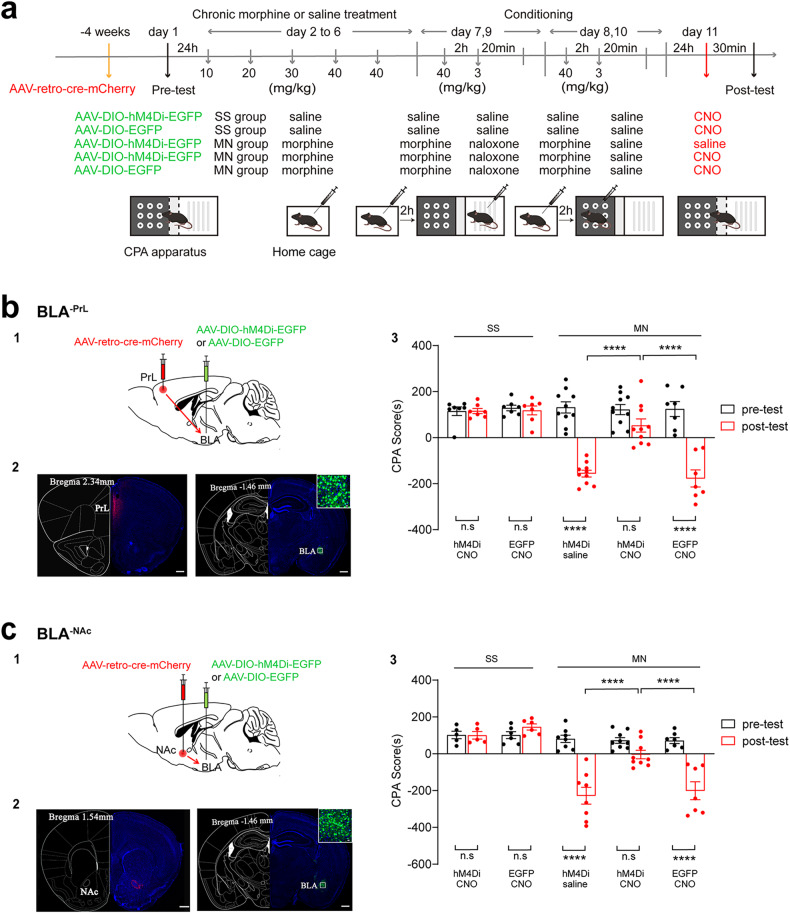
Effect of chemogenetic inhibition of BLA^-PrL^ or BLA^-NAc^ neurons on context-induced retrieval of morphine withdrawal memory. **a** Experimental timeline for CPA procedure. **b** Effect of chemogenetic inhibition of BLA^-PrL^ neurons on the CPA scores. **b1** Diagram of the injection of virus into the PrL and BLA. **b2** Left: anatomical locations of the injection site of cre-mCherry (red) in the PrL. Right: the expression of hM4Di-EGFP (green) in the BLA. Scale bar, 500 μm. **b3** Average CPA scores in SS-hM4Di-CNO (*n* = 7), SS-EGFP-CNO (*n* = 7), MN-hM4Di-Saline (*n* = 10), MN-hM4Di-CNO (*n* = 10), and MN-EGFP-CNO (*n* = 7) groups. Two-way ANOVA, ****P < 0.0001. **c** Effect of chemogenetic inhibition of BLA^-NAc^ neurons on the CPA scores. **c1** Diagram of the injection of virus into the NAc and BLA. **c2** Left: anatomical locations of the injection site of cre-mCherry (red) in the NAc. Right: the expression of hM4Di-EGFP (green) in the BLA. Scale bar, 500 μm. **c3** Average CPA scores in SS-hM4Di-CNO (*n* = 5), SS-EGFP-CNO (*n* = 6), MN-hM4Di-Saline (*n* = 8), MN-hM4Di-CNO (*n* = 9), and MN-EGFP-CNO (*n* = 7) groups. Two-way ANOVA, ****P < 0.0001. Means ± SEMs.

To further study the role of BLA^-PrL^ or BLA^-NAc^ neurons in conditioned context-induced retrieval of morphine withdrawal memory, we examined the influence of chemogenetic inhibition of BLA^-PrL^ or BLA^-NAc^ neurons on the CPA in morphine withdrawal mice. AAV-retro-cre-mCherry was infused into the PrL or the NAc in order to retrogradely deliver the cre-recombinase gene to the BLA. The cre dependent AAV-DIO-hM4Di-EGFP was injected in the BLA to allow for selective expression of hM4Di-EGFP in BLA^-PrL^ or BLA^-NAc^ neurons (Fig. 2b1, 2, c1, 2). Firstly, we examined the influence of chemogenetic inhibition of BLA^-PrL^ neurons on the CPA. The mice of five groups were subjected to the behavioral assay as shown in Fig. 2a. The results showed that in the MN group, the mice of the hM4Di-saline group and the EGFP-CNO group showed a strong aversion to the withdrawal-paired compartment, but the mice in the hM4Di-CNO group did not show a significant aversion to the withdrawal-paired compartment; in the SS group, the mice of the hM4Di-CNO group and the EGFP-CNO group did not show a significant aversion to the withdrawal-paired compartment (Two-way ANOVA, drug treatment factor, F _(4, 36)_ = 14.04, *P* < 0.0001; test condition factor, F _(1, 36)_ = 101.4, *P* < 0.0001; interaction factor, F _(4, 36)_ = 25.22, *P* < 0.0001; Bonferroni’s multiple comparisons, the pre-test vs. the post-test: in the SS group, hM4Di-CNO group, P > 0.9999; EGFP-CNO group, *P* > 0.9999; in the MN group, hM4Di-saline group, *P* < 0.0001; hM4Di-CNO group, P = 0.067; EGFP-CNO group, *P* < 0.0001. The post-test: hM4Di-CNO group vs. hM4Di-saline group, *P* < 0.0001; hM4Di-CNO group vs. EGFP-CNO group, *P* < 0.0001; hM4Di-saline group vs. EGFP-CNO group, *P* = 0.9697; Fig. 2b3). Then, we examined the influence of chemogenetic inhibition of BLA^-NAc^ neurons on the CPA. The results showed that in the MN group, the mice in the hM4Di-saline group and the EGFP-CNO group showed a strong aversion to the withdrawal-paired compartment, but the mice in the hM4Di-CNO group did not show a significant aversion to the withdrawal-paired compartment; in the SS group, the mice of the hM4Di-CNO group and the EGFP-CNO group did not show a significant aversion to the withdrawal-paired compartment (Two-way ANOVA, drug treatment factor, F _(4, 30)_ = 16.38, P < 0.0001; test condition factor, F _(1, 30)_ = 53.19, *P* < 0.0001; interaction factor, F _(4, 30)_ = 18.00, *P* < 0.0001. Bonferroni’s multiple comparisons, the pre-test vs. the post-test: in the SS group, hM4Di-CNO group, *P* > 0.9999; EGFP-CNO group, P > 0.9999; in the MN group, hM4Di-saline group, *P* < 0.0001; hM4Di-CNO group, *P* = 0.1252; EGFP-CNO group, *P* < 0.0001. The post-test: hM4Di-CNO group vs. hM4Di-saline group, P < 0.0001; hM4Di-CNO group vs. EGFP-CNO group, *P* < 0.0001; hM4Di-saline group vs. EGFP-CNO group, P = 0.956; Fig. 2c3). These results indicate that the activity of BLA^-PrL^ or BLA^-NAc^ neurons is necessary for context-induced retrieval of morphine withdrawal memory.

### BLA^-NAc^ neurons exhibit an increase in the expression of Arc and pERK, but BLA^-PrL^ neurons do not, during context-induced retrieval of morphine withdrawal memory

**Fig. 3:**
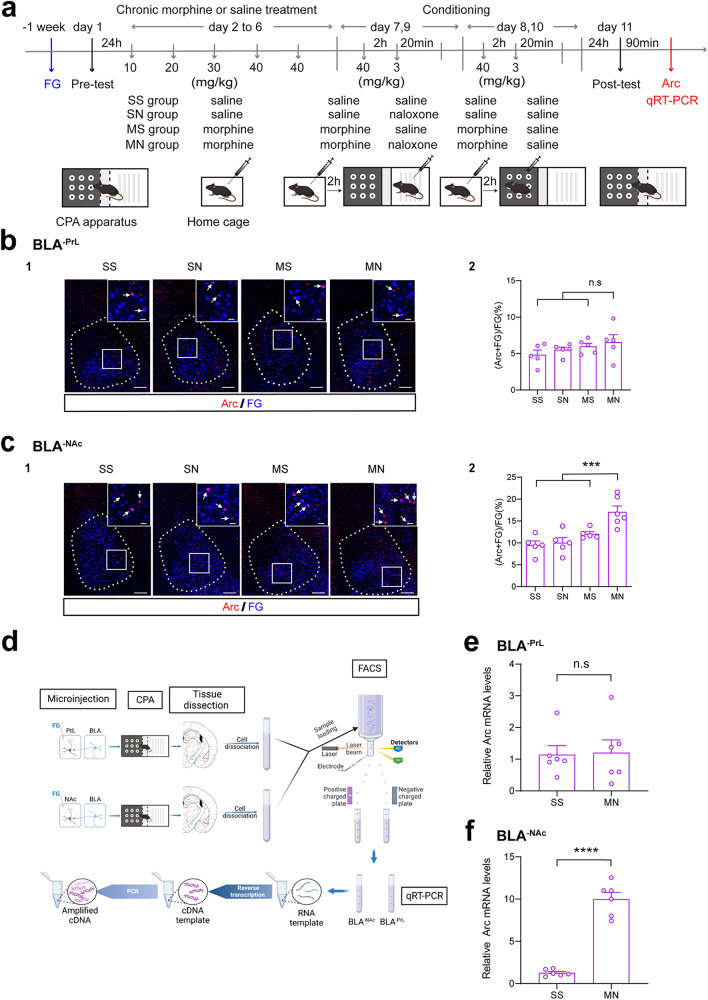
Effect of conditioned context on the expression of Arc in BLA^-PrL^ or BLA^-NAc^ neurons during context-induced retrieval of morphine withdrawal memory. **a** Experimental timeline for CPA procedure. **b** Effect of conditioned context on the expression of Arc in BLA^-PrL^ neurons during the retrieval of morphine withdrawal memory. **b1** Co-labeling neurons of Arc and FG in the BLA. Scale bar, 100 μm. Magnified image shows the boxed area. Scale bar, 20 μm. **b2** Quantification of co-labeling ratio of Arc and FG relative to FG in BLA^-PrL^ neurons (*n* = 5 mice in each group). One-way ANOVA, *P* > 0.05. **c** Effect of conditioned context on the expression of Arc in BLA^-NAc^ neurons during the retrieval of morphine withdrawal memory. **c1** Co-labeling neurons of Arc and FG in the BLA. Scale bar, 100 μm. Magnified image shows the boxed area. Scale bar, 20 μm. **c2** Quantification of co-labeling ratio of Arc and FG relative to FG in BLA^-NAc^ neurons (*n* = 5 mice in SS, SN, and MN groups; *n* = 6 mice in MS group). One-way ANOVA, ***P < 0.001. **d** Diagram of FACS and qRT-PCR of BLA^-PrL^ or BLA^-NAc^ neurons. **e** The relative Arc mRNA levels in FG-labeled BLA^-PrL^ neurons (*n* = 6 in each group from 12 mice, neurons from two mice were combined as one sample). Unpaired *t* test, *P* > 0.05. **f** The relative Arc mRNA levels in FG-labeled BLA^-NAc^ neurons (*n* = 6 in each group from 12 mice, neurons from two mice were combined as one sample). Unpaired *t* test, ****P < 0.0001. Means ± SEMs.

To study whether context induced a neural plasticity change in BLA^-PrL^ or BLA^-NAc^ neurons in morphine withdrawal mice, we examined the change in the expression of Arc, a marker of neuronal plasticity [17] after the exposure to context in BLA^-PrL^ or BLA^-NAc^ neurons in morphine withdrawal mice. The results showed that the average co-labeling percentage of Arc and FG relative to FG in BLA^-PrL^ neurons of the MN group had no significant difference compared to that of the SS group, the SN group and the MS group (One-way ANOVA, F _(3, 16)_ = 1.18, *P* > 0.05. Fig. 3b1, 2). However, context could significantly increase the expression of Arc in BLA^-NAc^ neurons in morphine withdrawal mice. The average co-labeling percentage of Arc and FG relative to FG of BLA^-NAc^ neurons in the MN group was significantly higher than that in the SS group, the SN group and the MS group (One-way ANOVA, F _(3, 17)_ = 10.59, *P* < 0.001. Fig. 3c1, 2). We also sorted out of BLA^-PrL^ or BLA^-NAc^ neurons by fluorescence-activated cell sorting (FACS) and performed qRT-PCR to examine the change in the expression of Arc mRNA in BLA^-PrL^ or BLA^-NAc^ neurons (Fig. 3d). As shown in supplementary Fig. S4a3 and Fig. S4b3, the mice in the MN group exhibited a strong aversion to the withdrawal-paired compartment, whereas mice in SS group did not exhibit a significant aversion to either compartment on matter in the mice of FG injection in PrL (Two-way ANOVA, drug treatment factor, F _(1, 22)_ = 29.17, *P* < 0.0001; test condition factor, F _(1, 22)_ = 60.46, *P* < 0.0001; interaction factor, F _(1, 22)_ = 66.89, *P* < 0.0001) and NAc (Two-way ANOVA, drug treatment factor, F _(1, 22)_ = 40.97, *P* < 0.0001; test condition factor, F _(1, 22)_ = 45.69, *P* < 0.0001; interaction factor, F _(1, 22)_ = 66.20, *P* < 0.0001). On this basis, qRT-PCR results of BLA^-PrL^ neurons isolated by FACS showed that the level of Arc mRNA in the MN group had no significant difference compared to that in the SS group (Unpaired *t* test, *P* > 0.05. Fig. 3e), whereas the level of Arc mRNA of BLA^-NAc^ neurons was significantly increased in the MN group compared to that in the SS group (Unpaired *t* test, *P* < 0.0001. Fig. 3f). These results suggest that context increases the expression of Arc in BLA^-NAc^ neurons, but does not in BLA^-PrL^ neurons during context-induced retrieval of morphine withdrawal memory.

**Fig. 4:**
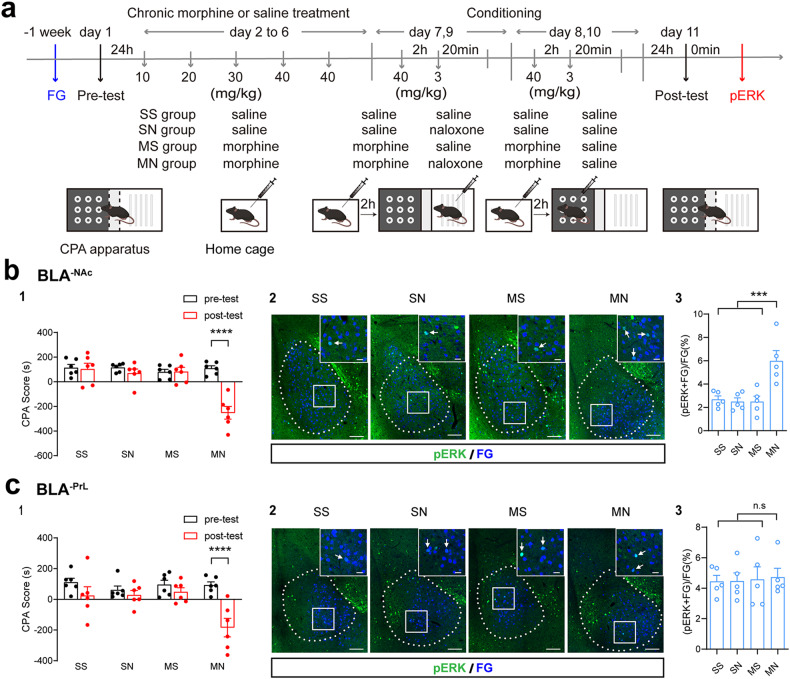
Effect of conditioned context on the expression of pERK in BLA^-PrL^ or BLA^-NAc^ neurons during context-induced retrieval of morphine withdrawal memory. **a** Experimental timeline for CPA procedure. **b** Effect of conditioned context on the expression of pERK in BLA^-NAc^ neurons during the retrieval of morphine withdrawal memory. **b1** Average CPA scores in SS (*n* = 6), SN (*n* = 6), MS (*n* = 6), and MN (*n* = 6) groups. Two-way ANOVA, ****P < 0.0001. **b2** Co-labeling neurons of pERK and FG in the BLA. Scale bar, 100 μm. Magnified image shows the boxed area. Scale bar, 20 μm. **b3** Quantification of co-labeling ratio of pERK and FG relative to FG in BLA^-NAc^ neurons (*n* = 5 mice in each group). One-way ANOVA, ***P < 0.001. **c** Effect of conditioned context on the expression of pERK in BLA^-PrL^ neurons during the retrieval of morphine withdrawal memory. **c1** Average CPA scores in SS (*n* = 6), SN (*n* = 6), MS (*n* = 6), and MN (*n* = 6) groups. Two-way ANOVA, ****P < 0.0001. **c2** Co-labeling neurons of pERK and FG in the BLA. Scale bar, 100 μm. Magnified image shows the boxed area. Scale bar, 20 μm. **c3** Quantification of co-labeling ratio of pERK and FG relative to FG in the BLA (*n* = 5 mice in each group). One-way ANOVA, *P* > 0.05. Means ± SEMs.

ERK is also an important molecule related to neural plasticity [23]. Moreover, it has been reported that ERK mediates the increase in the expression of Arc induced by brain-derived neurotrophic factor (BDNF) or cyclic adenosine monophosphate (cAMP) or long-term potentiation (LTP) [24–26]. Therefore, we examined the influence of context on the expression of pERK in BLA^-NAc^ neurons in morphine withdrawal mice. Mice experienced the CPA paradigm (Fig. 4a) after 1 week of recovery from the surgery of FG injection. The CPA results showed that the mice in the MN group exhibited a strong aversion to the withdrawal-paired compartment, whereas mice in other groups did not exhibit a significant aversion to either compartment (Two-way ANOVA, drug treatment factor, F _(3, 20)_ = 15.63, *P* < 0.0001; test condition factor, F _(1, 20)_ = 15.48, *P* < 0.001; interaction factor, F _(3, 21)_ = 22.48, *P* < 0.001. Fig. 4b1). After post-test, animals were sacrificed and slices containing the BLA were prepared. The co-labeling of pERK and FG was examined using immunofluorescence staining method. The results showed that after the context re-exposure, the co-labeling percentage of pERK and FG relative to FG in BLA^-NAc^ neurons was significantly increased in the MN group, but not in the SS, SN and MS groups (One-way ANOVA, F _(3, 16)_ = 9.7, *P* < 0.001. Fig. 4b2, 3). We also examined the influence of context on the expression of pERK in BLA^-PrL^ neurons using the mice which were injected with FG into the PrL. The CPA results showed that the MN group mice exhibited a strong aversion to the withdrawal-paired compartment, whereas mice in other groups did not exhibit a significant aversion to either compartment (Two-way ANOVA, drug treatment factor, F _(3, 20)_ = 3.86, *P* < 0.05; test condition factor, F _(1, 20)_ = 19.61, *P* < 0.001; interaction factor, F _(3, 20)_ = 5.06, *P* < 0.01. Fig. 4c1). After post-test, the animals were sacrificed and the slices containing the BLA were prepared. Immunofluorescence staining results showed that the co-labeling percentage of pERK and FG relative to FG in BLA^-PrL^ neurons in MN group had no significant difference compared with that of other three groups (One-way ANOVA, F _(3, 16)_ = 0.05, P > 0.05. Fig. 4c2, 3). To confirm the changes of ERK activity induced by context in BLA^-NAc^ and BLA^-PrL^ neurons in morphine withdrawal mice, we used another method (homogeneous time-resolved fluorescence (HTRF^®^) cell-based assay combining FACS) to examine the change in the expression of pERK in BLA^-PrL^ or BLA^-NAc^ neurons during context-induced retrieval of morphine withdrawal memory. As shown in supplementary Fig. S5a2 and Fig. S5b2, the mice in the MN group exhibited a strong aversion to the withdrawal-paired compartment, whereas mice in SS group did not exhibit a significant aversion to either compartment in the mice with FG injection in the NAc (Two-way ANOVA, drug treatment factor, F _(1,26)_ = 68.12, *P* < 0.0001; test condition factor, F _(1,28)_ = 113.1, *P* < 0.0001; interaction factor, F _(1,26)_ = 117.1, *P* < 0.0001) and the PrL (Two-way ANOVA, drug treatment factor, F _(1,26)_ = 45.50, *P* < 0.0001; test condition factor, F _(1,26)_ = 40.47, P < 0.0001; interaction factor, F _(1,26)_ = 51.19, *P* < 0.0001). On this basis, HTRF^®^ results of BLA^-NAc^ neurons isolated by FACS showed that the level of pERK in the MN group significantly increased compared to that in the SS group (Paired *t* test, *P* < 0.01. Supplementary Fig. S5a3), but the level of pERK of BLA^-PrL^ neurons in the MN group had no significant difference compared to that in the SS group (Paired *t* test, *P* > 0.05. Fig. S5b3). These results suggest that context increases the expression of pERK in BLA^-NAc^ neurons, but does not in BLA^-PrL^ neurons during context-induced retrieval of morphine withdrawal memory.

### pERK was the upstream molecule of Arc, whereas D1 receptor was the upstream molecule of pERK in BLA^-NAc^ neurons during context-induced retrieval of morphine withdrawal memory

**Fig. 5:**
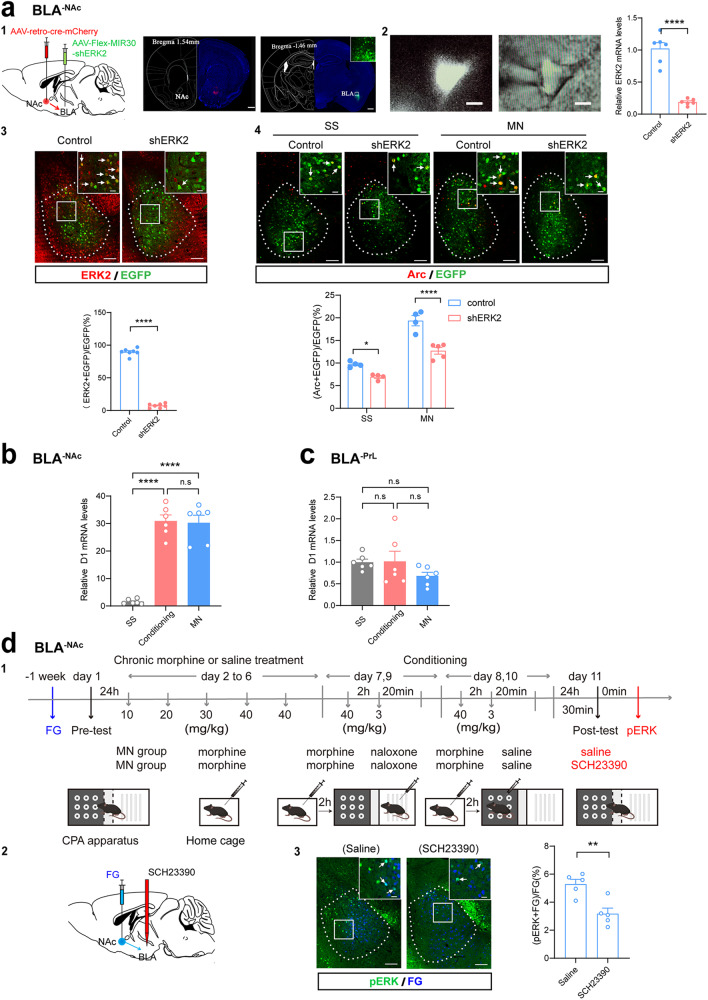
Relationship of Arc, pERK and D1 receptors in BLA^-NAc^ neurons during context-induced retrieval of morphine withdrawal memory. **a1** Left: diagram of the injection of virus into the BLA and NAc. Middle: anatomical locations of the injection site of cre-mCherry (red) in the NAc. Right: the expression of MIR30-shERK2-EGFP (green) in the BLA. Scale bar, 500 μm. **a2** Left: BLA^-NAc^ neurons labeled with fluorescent microspheres and harvesting the cytoplasmic contents of the labeled neuron via a patch pipette under visual control. Scale bar, 5 μm. Right: the averaged ERK2 mRNA expression level in microsphere-labeled neurons in the BLA in the control and shERK2 groups by qRT-PCR. Unpaired *t* test, ****P < 0.0001. **a3** Top: co-labeling neurons of ERK2 and EGFP in the BLA. Scale bar, 100 μm. Magnified image shows the boxed area. Scale bar, 20 μm. Bottom: Quantification of co-labeling ratio of ERK2 and EGFP relative to EGFP in the BLA (*n* = 7 mice in each group). Unpaired *t* test, ****P < 0.0001. **a4** Top: co-expression of Arc and EGFP in the BLA^-NAc^ neurons in different groups. Scale bar, 100 μm. Magnified image shows the boxed area. Scale bar, 20 μm. Bottom: quantification of co-labeling ratio of Arc and EGFP relative to EGFP in BLA^-NAc^ neurons in different groups (*n* = 4 in SS (control) and *n* = 4 SS (shERK2) group; *n* = 4 in MN (control) group and *n* = 5 in MN (shERK2) group). Two-way ANOVA, *P < 0.05, ****P < 0.0001. **b** The relative D1 mRNA levels in BLA^-NAc^ neurons (*n* = 6 in each group from 12 mice, neurons from two mice were combined as one sample). One-way ANOVA, ****P < 0.0001. **c** The relative D1 mRNA levels in BLA^-PrL^ neurons (*n* = 6 in each group from 12 mice, neurons from two mice were combined as one sample). One-way ANOVA, *P* > 0.05. **d1** Experimental timeline for CPA procedure. **d2** Diagram of the injection of fluorogold (FG) into the PrL and the implantation of annular tube into the BLA. **d3** Left: co-labeling neurons of pERK and FG in the BLA. Scale bar, 100 μm. Magnified image shows the boxed area. Scale bar, 20 μm. Right: quantification of co-labeling ratio of pERK and FG relative to FG in the BLA (*n* = 5 mice in MN (saline) and MN (SCH23390) groups). Unpaired *t* test, **P < 0.01.

The above results showed that there was an increase in the expression of Arc and pERK in BLA^-NAc^ neurons during context-induced retrieval of morphine withdrawal memory, but the causal relationship between them in this process is unknown. To answer this question, we examined the influence of the inhibition of ERK expression in BLA^-NAc^ neurons on context-induced increase in the expression of Arc in BLA^-NAc^ neurons during retrieval of morphine withdrawal memory. ERK has two major isoforms: ERK1 and ERK2 [27, 28]. Since there are some evidences suggesting that ERK may be upstream molecule of Arc [29, 30], here, firstly, we examined the influence of ERK2 mRNA interference on context-induced increase of Arc expression in BLA^-NAc^ neurons. The cre-dependent MIR30-shERK2 (short-hairpin RNA of ERK2)-EGFP virus (shERK2 group) or the same viral vector carrying only EGFP (control group) was injected into the BLA and AAV-retro-cre virus was injected into the NAc to specifically control the expression of short-hairpin RNA of ERK2 in BLA^-NAc^ neurons. The shERK2 were allowed to express at least 4 weeks and the expressed EGFP in the BLA was shown in the right panel of Fig. 5a1. The inhibitory efficiency of RNA interference virus on ERK2 mRNA expression in BLA^-NAc^ neurons was examined by using qRT-PCR method. The left panel of Fig. 5a2 shows an EGFP positive BLA^-NAc^ neuron and its cytoplasmic content was harvested by a patch pipette to detect ERK2 mRNA using single-cell RT-PCR kit. The result showed that the ERK2 mRNA was significantly decreased in the BLA^-NAc^ neurons in shERK2 group compared with control group (Unpaired *t* test, *P* < 0.0001. Right panel of Fig. 5a2). Immunofluorescence staining analysis also showed a reduction in ERK2 protein expression in shERK2 group. The percentage of co-labeling ERK2 and EGFP relative to EGFP in BLA^-NAc^ neurons in the shERK2 group was significantly lower than that in the control group (Unpaired *t* test, *P* < 0.0001. Bottom panel of Fig. 5a3). On this basis, we firstly examined the influence of ERK2 interference in BLA^-NAc^ neurons on CPA and then on context-induced increase in Arc expression during retrieval of morphine withdrawal memory. The mice of SS group and MN group were divided into the EGFP expressing (control) or MIR30-shERK2-EGFP expressing (shERK2) mice. The results showed that the mice of SS (control) group and SS (shERK2) group did not exhibit a significant aversion to either compartment. The mice of MN (control) group had a strong aversion to the withdrawal-paired compartment, but the mice of MN (shERK2) group had not a significant aversion to the withdrawal-paired compartment after ERK2 expression were reduced by RNA interference (Two-way ANOVA, drug treatment factor, F _(3, 24)_ = 11.74, *P* < 0.0001; test condition factor, F _(1, 24)_ = 5.16, *P* < 0.05; interaction factor, F _(3, 24)_ = 19.71, *P* < 0.0001. Bonferroni’s multiple comparisons: the pre-test vs. the post-test in SS (control) (*P* = 0.2875), SS (shERK2) (*P* = 0.0790), MN (control) (*P* < 0.0001) and MN (shERK2) (*P* = 0.2542) groups. The post-test of SS (control) group vs. SS (shERK2) group: *P* = 0.9964; MN (control) vs. MN (shERK2) group: *P* < 0.0001. Supplementary Fig. S6a3). Ninety minutes after post-test, the animals were sacrificed and the expressions of Arc in BLA^-NAc^ neurons in the SS group and the MN group were examined by immunofluorescent staining method. The results showed that Arc expression of BLA^-NAc^ neurons was significantly increased in the MN (control) group compared to the SS (control) group, but Arc expression of BLA^-NAc^ neurons was significantly reduced in the MN (shERK2) group compared to the MN (control) group, and the expression level of Arc in MN (shERK2) group was close to that of the SS (control) group (Two-way ANOVA, drug treatment factor, F _(1, 13)_ = 114.7, *P* < 0.0001; shERK2 interference factor, F _(1, 13)_ = 42.81, *P* < 0.0001; interaction factor, F _(1, 13)_ = 7.24, *P* < 0.05. Bonferroni’s multiple comparisons: the control vs. the shERK2 in MN groups, *P* < 0.0001. Fig. 5a4). These results suggest that ERK2 in BLA^-NAc^ neurons participates in context-induced retrieval of morphine withdrawal memory and is an upstream molecule for the increase in the expression of Arc in the BLA^-NAc^ neurons during context-induced retrieval of morphine withdrawal memory.

Then, we examined the influence of ERK1 knockdown in BLA^-NAc^ neurons on CPA. The results showed that the mice of SS (control) group and SS (shERK1) group did not exhibit a significant aversion to either compartment. The mice of MN (control) group and MN (shERK1) both had a strong aversion to the withdrawal-paired compartment (Two-way ANOVA, drug treatment factor, F _(3, 28)_ = 6.36, *P* < 0.01; test condition factor, F _(1, 28)_ = 22.61, *P* < 0.0001; interaction factor, F _(3, 28)_ = 7.81, *P* < 0.001. Bonferroni’s multiple comparisons: the pre-test vs. the post-test in SS (control) (*P* = 0.4009), SS (shERK1) (*P* = 0.8335), MN (control) (*P* < 0.001) and MN (shERK1) (*P* < 0.0001) groups. The post-test of SS (control) group vs. SS (shERK1) group: *P* = 0.0928; MN (control) vs. MN (shERK1) group: *P* = 0.9998. Supplementary Fig. S6b3). This result suggests that ERK1 in BLA^-NAc^ neurons does not participate in context-induced retrieval of morphine withdrawal memory.

Several lines of evidence suggest that dopamine D1 receptor is an important upstream molecular that activates ERK [31–33]. Moreover, our previous study showed that the intra-BLA injection of D1 receptor antagonist canceled context-induced retrieval of morphine withdrawal memory [34], suggesting that D1 receptors in the BLA participated in context-induced retrieval of morphine withdrawal memory. However, for different BLA projection neurons, which projection neurons are modulated by D1 receptors remain unknown. Here, using BLA^-PrL^ or BLA^-NAc^ neurons sorted in the Arc mRNA expression experiment (Fig. 3d), we examined the expression of D1 receptors in BLA^-PrL^ or BLA^-NAc^ neurons after withdrawal-context conditioning and the retrieval morphine withdrawal memory. In the withdrawal-context conditioning group, the results from qRT-PCR showed that D1 mRNA level was significantly increased after withdrawal-context conditioning, compared to the SS group (One-way ANOVA, F _(2, 15)_ = 5.74, P < 0.05. Fig. 5b) in BLA^-NAc^ neurons, but did not in BLA^-PrL^ neurons (One-way ANOVA, F _(2, 15)_ = 0.33, *P* > 0.05. Fig. 5c). In the morphine withdrawal memory retrieval group, the results from qRT-PCR showed that D1 mRNA level was significantly increased after morphine withdrawal memory retrieval, compared to the SS group (One-way ANOVA, F _(2, 15)_ = 5.74, *P* < 0.05. Fig. 5b) in BLA^-NAc^ neurons, but did not in BLA^-PrL^ neurons (One-way ANOVA, F _(2, 15)_ = 0.33, *P* > 0.05. Fig. 5c). These results suggest that before the retrieval of morphine withdrawal memory, the expression of D1 receptors mRNA in BLA^-NAc^ neurons is already at a high level due to withdrawal-context conditioning in BLA^-NAc^ neurons, but does not in BLA^-PrL^ neurons.

To study whether D1 receptor is an upstream molecule of ERK activation during context-induced retrieval of morphine withdrawal memory, we examined the influence of D1 receptor inhibitor on context-induced increase of pERK in BLA^-NAc^ neurons in morphine withdrawal mice. The morphine dependent mice were divided into two groups randomly: the saline group that was injected saline into the BLA through a cannula at 30 min before post-test; the SCH23390 group that was injected SCH23390 into the BLA through a cannula at 30 min before the post-test. One week after FG injection, mice were subjected to behavioral training as illustrated in Fig. 5d1. The results showed that the CPA score of the mice in SCH23390 group was significantly decreased compared with that of saline group (Two-way ANOVA, drug treatment factor, F _(1, 10)_ = 10.61, *P* < 0.01; test condition factor, F _(1, 10)_ = 51.62, *P* < 0.0001; interaction factor, F _(1, 10)_ = 15.76, *P* < 0.01. Bonferroni’s multiple comparisons: the pre-test vs. the post-test in saline (P < 0.0001) and SCH23390 (*P* = 0.0926) groups. The post-test of saline group vs. SCH23390 group: *P* < 0.001. Supplementary Fig. S7a2). After post-test, the animals were sacrificed and the expressions of pERK in different groups were examined. The result showed that the co-labeling percentage of pERK and FG relative to FG in BLA^-NAc^ neurons in the SCH23390 group was significantly lower than that in the saline group (Unpaired *t* test, *P* < 0.01. Fig. 5d2). This result suggests that D1 receptor is an upstream molecule of ERK activation during context-induced retrieval of morphine withdrawal memory.

We also studied the role of D2 receptors of the BLA in the context-induced retrieval of morphine withdrawal memory by examining the influence of D2 receptor antagonist sulpiride [35] on context-induced retrieval of morphine withdrawal memory. The morphine dependent mice were divided into two groups randomly: saline group where mice were injected with saline into the BLA through a cannula at 10 min before post-test; sulpiride group where mice were injected with sulpiride into the BLA through a cannula at 10 min before the post-test. One week after the surgery, mice were subjected to behavioral training as illustrated in Fig. 5d1. The results showed that the mice in saline group and sulpiride group showed a strong aversion to the withdrawal-paired compartment (Two-way ANOVA, drug treatment factor, F _(1, 15)_ = 0.1982, *P* = 0.6626; test condition factor, F _(1, 15)_ = 192.6, *P* < 0.0001; interaction factor, F _(1, 15)_ = 0.0098, *P* = 0.9224. Bonferroni’s multiple comparisons: the pre-test vs. the post-test in saline (*P* < 0.0001) and sulpiride (*P* < 0.0001) groups. The post-test of saline group vs. sulpiride group: *P* > 0.9999. Supplementary Fig. S7b2). This result suggests that D2 receptors in the BLA do not participate in the context-induced retrieval of morphine withdrawal memory.

### D1 receptors strengthen AMPA receptor mediated-currents via pERK in BLA^-NAc^ neurons during context-induced retrieval of morphine withdrawal memory

**Fig. 6:**
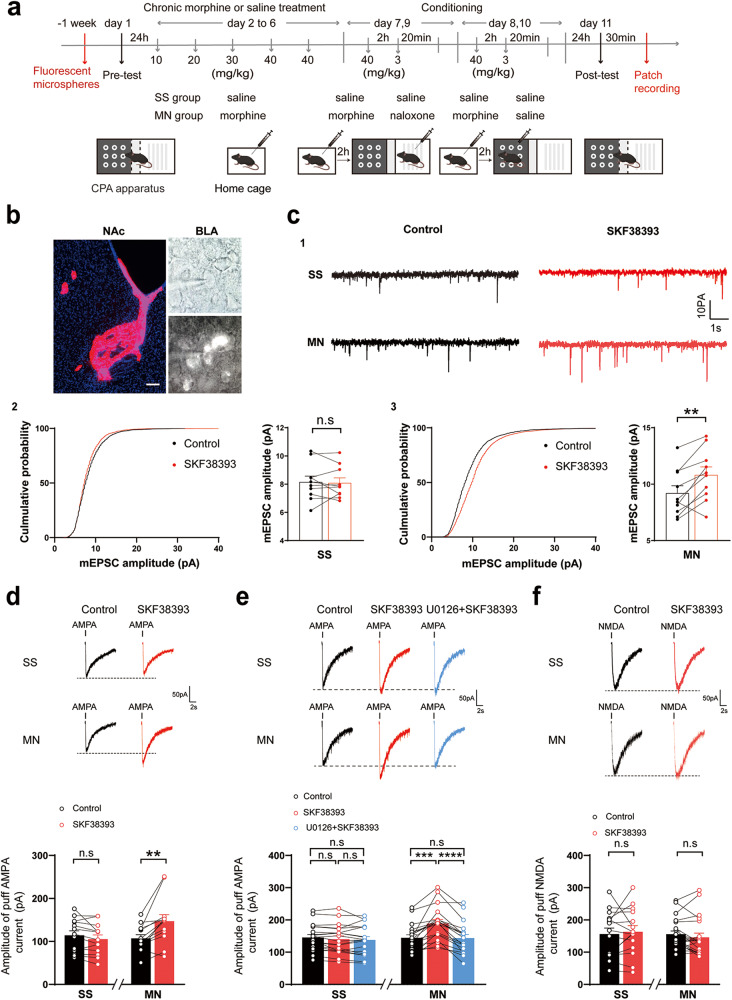
Effect of D1 receptor activation on AMPA and NMDA receptor functions in BLA^-NAc^ neurons during context-induced retrieval of morphine withdrawal memory. **a** Experimental timeline for CPA procedure. **b** Left: the injection site of the fluorescent microsphere (red) in the NAc. Scale bar, 100 μm. Right: the fluorescent image of BLA^-NAc^ neurons labeled with fluorescent microsphere retrogradely transported from the NAc in a brain slice and its image under infrared differential interference contrast (IR-DIC) microscopy. **c1** Top: Representative traces of mEPSCs before (Control) and after SKF38393 treatment in BLA^-NAc^ neurons in SS and MN group. Holding potential: −70 mV. **c2** Left: Cumulative probability of the amplitude. Right: mEPSCs amplitude of Control vs. SKF38393 in BLA^-NAc^ neurons in SS (*n* = 10 cells from 6 mice) groups. Paired *t* test, *P* > 0.05. **c3** Left: Cumulative probability of the amplitude. Right: mEPSCs amplitude of Control vs. SKF38393 in BLA^-NAc^ neurons in MN (*n* = 10 cells from 5 mice) groups. Paired *t* test, **P < 0.01. **d** Top: representative traces of puff-AMPA-elicited currents before (Control) and after SKF38393 treatment in BLA^-NAc^ neurons in SS and MN group. Bottom: bar graphs show the effect of D1 receptor agonist SKF38393 on puff-AMPA-elicited currents of labeled BLA^-NAc^ neurons recorded from SS (*n* = 13 cells from 8 mice) and MN (*n* = 13 cells from 8 mice) groups. Paired *t* test, **P < 0.01. **e** Top: representative traces of of puff-AMPA-elicited currents before (Control) and after SKF38393, U0126 + SKF38393 treatment in BLA^-NAc^ neurons in SS and MN group. Bottom: bar graphs show the effect of D1 receptor agonist SKF38393 and inhibitor of ERK/MAPK activation U0126 on puff-AMPA-elicited currents of labeled BLA^-NAc^ neurons recorded from SS (*n* = 19 cells from 8 mice) and MN (*n* = 19 cells from 9 mice) groups. RM one-way ANOVA, ***P < 0.001, ****P < 0.0001. **f** Top: representative traces of puff-NMDA-elicited currents before (Control) and after SKF38393 treatment in BLA^-NAc^ neurons in SS and MN group. Bottom: bar graphs show the effect of D1 receptor agonist SKF38393 on puff-NMDA-elicited currents of labeled BLA^-NAc^ neurons recorded from SS (*n* = 15 cells from 7 mice) and MN (*n* = 21 cells from 8 mice) groups. Paired *t* test, *P* > 0.05. Means ± SEMs.

BLA-^NAc^ neurons require to be excited by glutamatergic input in order to participate in context-induced retrieval of morphine withdrawal memory. Here, we examined whether D1 receptor pathway had a strengthening effect on glutamatergic input to BLA^-NAc^ neurons. Seven days after the injection of microsphere into the NAc, mice were subjected to behavioral training as illustrated in Fig. 6a. Thirty minutes after post-test, the mice were sacrificed and brain slices were collected for electrophysiological experiments. Figure 6b showed the image of BLA^-NAc^ neurons labeled by microsphere. Firstly, we examined the effect of D1 receptor agonist SKF38393 (10 μM) on the mEPSCs of BLA^-NAc^ neurons in the SS or MN group. The mice in the MN group exhibited a strong aversion to the withdrawal-paired compartment, whereas mice in SS group did not exhibit a significant aversion to either compartment (Two-way ANOVA, drug treatment factor, F _(1, 9)_ = 31.33, *P* < 0.001; test condition factor, F _(1, 9)_ = 163.9, *P* < 0.0001; interaction factor, F _(1, 9)_ = 38.93, *P* < 0.001. Supplementary Fig. S8a2). Figure 6C1 illustrates typical raw current traces before and after SKF38393 in the SS and MN groups. The result showed that SKF38393 had no significant effect on the amplitude of mEPSCs in the SS group, the average amplitude of mEPSCs was 8.16 ± 0.41 pA before and 8.09 ± 0.36 pA after SKF38393 (Paired *t* test, *P* > 0.05; Fig. 6c2). But in the MN group, SKF38393 could significantly increase the amplitude of mEPSCs, the average amplitude of mEPSCs increased from 9.20 ± 0.65 pA before to 10.81 ± 0.72 pA after SKF38393 (Paired *t* test, *P* < 0.01; Fig. 6c3) and the average percentage of increase was 18.67 ± 5.20%.

To directly detect the effect of D1 receptors on postsynaptic AMPA receptor activity, we examined the effect of SKF38393 on AMPA receptor currents which were elicited by puff application of AMPA to BLA^-NAc^ neurons in the SS or MN group. The mice in the MN group exhibited a strong aversion to the withdrawal-paired compartment, whereas mice in SS group did not exhibit a significant aversion to either compartment (Two-way ANOVA, drug treatment factor, F _(1, 14)_ = 75.90, *P* < 0.0001; test condition factor, F _(1, 14)_ = 71.24, *P* < 0.0001; interaction factor, F _(1, 14)_ = 76.14, P < 0.0001. Supplementary Fig. S8b2). Representative raw traces showed that puff application of 10 μM AMPA induces an inward current in both groups (Top panel of Fig. 6d). From original recordings, we could see that SKF38393 had no significant effect on the amplitude of evoked AMPA-EPSCs in the SS group, the average amplitude of evoked AMPA-EPSCs was 114.1 ± 10.66 pA before and 105.3 ± 9.88 pA after SKF38393 (Paired t test, P > 0.05; Bottom panel of Fig. 6d). But in the MN group, SKF38393 could significantly increase the amplitude of evoked AMPA-EPSCs, the average amplitude of evoked AMPA-EPSCs increased from 107.2 ± 8.40 pA before to 147.2 ± 15.04 pA after SKF38393 (Paired *t* test, *P* < 0.01; Bottom panel of Fig. 6d) and the average percentage of increase was 37.55 ± 8.86%. We further studied whether D1 receptors-induced enhancement of AMPA receptor function in BLA^-NAc^ neurons was via ERK by examining the effect of ERK inhibitor U0126 [36] on SKF38393-induced increase in AMPA-EPSCs. The mice in the MN group exhibited a strong aversion to the withdrawal-paired compartment, whereas mice in SS group did not exhibit a significant aversion to either compartment (Two-way ANOVA, drug treatment factor, F _(1, 15)_ = 133.5, *P* < 0.0001; test condition factor, F _(1, 15)_ = 60.55, *P* < 0.0001; interaction factor, F _(1, 15)_ = 50.46, *P* < 0.0001. Supplementary Fig. S8c2). As shown in typical raw traces (Top panel of Fig. 6e), SKF38393-induced increase in evoked AMPA-EPSCs of BLA^-NAc^ neurons disappeared in the presence of U0126 (10 µM) in the MN group. In the SS group, the average amplitude of AMPA-EPSCs was 145.50 ± 9.28 pA before and 140.3 ± 10.23 pA after SKF38393, and it was 138.20 ± 10.37 pA in the presence of SKF38393 and U0126 (One-way ANOVA, F _(2, 36)_ = 2.96, *P* > 0.05: Control group vs. SKF38393 group, *P* > 0.05; Control group vs. U0126 + SKF38393 group, *P* > 0.05; SKF38393 group vs. U0126 + SKF38393 group, *P* > 0.05; Bottom panel of Fig. 6e). In the MN group, the average amplitude of AMPA-EPSCs was 144.6 ± 9.59 pA before and 189.20 ± 11.92 pA after SKF38393, while it was 143.7 ± 11.28 pA in the presence of SKF38393 and U0126 (One-way ANOVA, F _(1.50, 27.04)_ = 31.11, *P* < 0.0001: Control group vs. SKF38393 group, *P* < 0.001; Control group vs. U0126 + SKF38393 group, *P* > 0.05; SKF38393 group vs. U0126 + SKF38393 group, *P* < 0.0001; Bottom panel of Fig. 6e).

We also examined the effect of SKF38393 on NMDA receptor currents which were elicited by puff administration of NMDA directly to BLA^-NAc^ neurons in the SS or MN group. The mice in the MN group exhibited a strong aversion to the withdrawal-paired compartment, whereas mice in SS group did not exhibit a significant aversion to either compartment (Two-way ANOVA, drug treatment factor, F _(1, 13)_ = 97.02, *P* < 0.0001; test condition factor, F _(1, 13)_ = 91.66, *P* < 0.0001; interaction factor, F _(1, 13)_ = 96.89, *P* < 0.0001. Supplementary Fig. S8d2). Representative raw traces showed that puff application of 100 μM NMDA induces an inward current in both groups (Top panel of Fig. 6f). From original recordings, we could see that SKF38393 had no significant effect on the amplitude of evoked NMPA-EPSCs in the both groups. In the SS group, the average amplitude of evoked NMPA-EPSCs was 156.20 ± 18.46 pA before and 163.60 ± 18.57 pA after SKF38393 (Paired t test, *P* > 0.05. Bottom panel of Fig. 6f). In the MN group, the average amplitude of evoked NMPA-EPSCs was 155.80 ± 10.09 pA before and 146.20 ± 12.85 pA after SKF38393 (Paired *t* test, *P* > 0.05. Bottom panel of Fig. 6f). These results suggest that D1 receptors can strengthen AMPA receptor-mediated glutamatergic input to BLA^-NAc^ neurons via pERK, but has no effect on NMDA receptor-mediated glutamatergic input to BLA^-NAc^ neurons during context-induced retrieval of morphine withdrawal memory.

## Discussion

The main findings of the present study were that (1) although both glutamatergic BLA^-PrL^ and BLA^-NAc^ neurons participated in context-induced retrieval of morphine withdrawal memory, they exhibited a different change in the expression of some molecules: there was an increase in the expression of Arc and pERK in BLA^-NAc^ neurons, but not in BLA^-PrL^ neurons during context-induced retrieval of morphine withdrawal memory; (2) pERK was the upstream molecule of Arc, whereas D1 receptor was the upstream molecule of pERK in BLA^-NAc^ neurons during context-induced retrieval of morphine withdrawal memory; (3) D1 receptors also strengthened AMPA receptors, but not NMDA receptors, -mediated glutamatergic input to BLA^-NAc^ neurons via pERK during context-induced retrieval of morphine withdrawal memory.

The BLA is composed of excitatory projection neurons (80–90%) and inhibitory interneurons (10–20%), with the projection neurons mediating BLA’s communication with other brain regions under fine-tuning of interneurons [37]. BLA^-PrL^ and BLA^-NAc^ neurons are BLA excitatory projection neurons, but they project to different brain regions. The present study found that these two kinds of BLA neurons that projected to different extra-amygdala regions participated in the same task, that is, the retrieval of morphine withdrawal memory, indicating that the long-range connectivity of the BLA mediated by BLA^-PrL^ and BLA^-NAc^ neurons encoded the retrieval of morphine withdrawal memory.

Previous studies showed that chemogenetic or optogenetic inhibition of BLA^-PrL^ neurons reduced fear response, whereas photostimulation of BLA^-PrL^ neurons increased fear response [38]. The present study showed that context-induced activation of BLA^-PrL^ neurons participated in the retrieval of morphine withdrawal memory. These evidences indicate that when persons with substance dependence disorders encounter context that is previously associated with drug withdrawal symptoms, context-induced activation of BLA^-PrL^ neurons, on one hand, induces the retrieval of drug withdrawal memory and, on the other hand, increases fear response, which may cooperate with the retrieval of morphine withdrawal memory to promote drug relapse. This statement is consistent with the fact that individuals with context induced-fear response are more likely to relapse to drug use when context associated with drug-seeking is encountered [39].

It has been known that BLA^-NAc^ neurons play an important role in reward-related behaviors [40–43]. It is worth noting that BLA^-NAc^ neurons also are involved in aversive behaviors [44, 45]. The basis for BLA^-NAc^ neurons to mediate positive and negative behavior may be related to heterogeneous populations of BLA^-NAc^ neurons. Joshua Kim et al reported that BLA^-NAc^ neurons in anterior BLA represented negative valence, while BLA^-NAc^ neurons in posterior BLA represented positive valence [46]. Therefore, if activating BLA^-NAc^ neurons in anterior BLA, it should induce aversive behaviors, which was supported by the result from Xian Zhang et al [45] and by our present result, whereas if activating BLA^-NAc^ neurons in posterior BLA, it should induce reward-related behaviors.

Within the large-scale neuronal networks encoding morphine withdrawal memory, how different projection neurons participate in the retrieval of morphine withdrawal memory is poorly understood. There are two possible ways of different projection neurons participating in the retrieval of morphine withdrawal memory: one is that after being activated, they are only a pathway that mediates the passage of signals to induce a retrieval of memory signals at other projection neurons and another is that they exhibit a retrieval of memory signals by themselves. To test this hypothesis, we examined the influence of context on the expression of c-Fos, a marker of neuronal activation [22], and Arc, a marker of neuronal plasticity [17]. Arc not only participates in memory formation [47], but also is involved in the retrieval of memory [48]. Arc knockout mice failed to form long-lasting memories [17] and the inhibition of Arc expression disrupted the retrieval of memory [49]. Therefore, the results that context induces an increase in the expression of c-Fos in both BLA^-PrL^ and BLA^-NAc^ neurons, but only increases the expression of Arc in BLA^-NAc^ neurons, suggest that after being activated, BLA^-PrL^ neurons may be only a pathway that mediates the passage of signals to induce a retrieval of memory signals at other downstream projection neurons, whereas BLA^-NAc^ neurons may exhibit a retrieval of memory signals by themselves. Our previous result that BLA^-PrL^ neurons innervated another kind of BLA projection neurons through a feedback circuit from the PrL to the BLA [12] suggested that this other downstream projection neuron of BLA^-PrL^ neurons might be BLA^-NAc^ neurons where they exhibited a retrieval of memory signals.

In addition to Arc, ERK is also an important molecule related to neural plasticity and memory [23]. ERK activity has been shown to be necessary for the formation of memory in several different animals and training paradigms [50]. In rats, both cued and contextual fear conditioning were found to result in the activation of ERK in the hippocampus, and the inhibition of ERK prevented memory formation [51]. ERK is an excellent candidate for regulators of synaptic plasticity [52], a putative mechanism for the neuronal basis of memory. ERK are expressed in dendrites and somas of pyramidal neurons of the adult nervous system [53]. ERK can be activated by several neurotransmitters [54]. Substrates of ERK regulate processes thought to be important for synaptic plasticity, including second messenger generation, cytoskeletal modulation, and transcription [55]. The LTP, which is one of the cellular mechanisms involved in memory [56], also requires the activation of ERK [57, 58]. Therefore, the result that context induces an increase in the expression of ERK in BLA^-NAc^ neurons, but has no effect on the expression of ERK in BLA^-PrL^ neurons further supports that after being activated, BLA^-NAc^ neurons may exhibit a retrieval of memory signals by themselves, whereas BLA^-PrL^ neurons may be only a pathway that mediates the passage of signals to induce a retrieval of memory signals at other projection neurons. Moreover, our results showed that in BLA^-NAc^ neurons, the activation of ERK was an upstream mechanism of context-induced increase in the expression of Arc. This result is consistent with the reports that Arc induction requires ERK activation [24–26].

ERK1 and ERK2 are two major isoforms of ERK [27, 28]. Although they have 90% sequence identity, ERK 1 and ERK2 appear to have distinct functional effects [27]. Boulton et al reported that ERK2 was specifically expressed in neurons by comparing levels of ERK1 and ERK2 in whole brain and cultured glial cells [59]. Immunohistochemical and electron-microscopic studies by Fiore et al revealed that prominent ERK2 staining was in neuronal cell bodies and dendrites, and ERK2 in dendrites was closely associated with microtubules [53]. When neurons were excited, the ERK2 pathway was activated to a plasticity-permissive state, both at the level of dendrites and by regulating nuclear transcription [27]. Therefore, it is most likely that ERK2 in BLA^-NAc^ neurons is an upstream molecule that induces an increased expression of Arc and participate in the retrieval of morphine withdrawal memory. This hypothesis is supported by our present results that the inhibition of ERK2 can significantly inhibit context-induced increase in the expression of Arc and context-induced retrieval of morphine withdrawal memory.

Among various receptors that may activate ERK, several lines of evidence suggest that D1 receptor may be important one. Borgkvist et al reported that context-induced ERK activation in withdrawn mice was abolished by pre-treatment with the specific D1 receptor antagonist SCH23390 [31]. D1 receptor antagonist SKF81297 could increase phosphorylation of ERK in the granule cells of the dentate gyrus and this effect was prevented by genetic inactivation of D1 receptor [32]. The binding of dopamine to D1 receptors activated PKA to phosphorylate Rasgrp2, which was followed by Rap1 activation, leading to the activation of ERK [33]. Moreover, our previous study showed that the intra-BLA injection of D1 receptor antagonist canceled context-induced retrieval of morphine withdrawal memory [34]. However, among various BLA projection neurons, which projection neurons are modulated by dopamine D1 receptors remain unknown. The present result showed that before the memory retrieval, the expression of D1 receptors in context-withdrawal conditioning group significantly increased in BLA^-NAc^ neurons, but did not in BLA^-PrL^ neurons, and the intra-BLA injection of D1 receptor antagonist significantly decreased context-induced activation of ERK in BLA^-NAc^ neurons. These results indicated that BLA^-NAc^ neurons were modulated more by dopamine D1 receptors and the activation of D1 receptors was an upstream molecular event of the activation of ERK in BLA^-NAc^ neurons during context-induced retrieval of morphine withdrawal memory.

The BLA is densely innervated by glutamatergic projections from other brain regions [60]. Exposure to an aversive-conditioned stimulus induced an augmentation of glutamate signaling within the BLA [61, 62]. During memory retrieval, behavioral expression was impaired by intra-amygdala blockade of AMPA receptors [62]. However, during context-induced retrieval of morphine withdrawal memory, whether glutamate signaling is modulated by dopamine D1 receptors in BLA^-NAc^ neurons remains unknown. The present result that the activation of D1 receptors can strengthen AMPA receptor-mediated glutamatergic input to BLA^-NAc^ neurons via ERK suggests that in addition to the induction of an increased Arc expression, D1-ERK pathway also augments AMPA receptor-mediated glutamatergic input to BLA^-NAc^ neurons during context-induced retrieval of morphine withdrawal memory.

One theoretical implication of the present findings is that although two different BLA projection neurons (BLA^-PrL^ and BLA^-NAc^ neurons) are involved in context-induced retrieval of morphine withdrawal memory, BLA^-NAc^ neurons exhibit changes in molecules related to neural plasticity, such as Arc and ERK, but BLA^-PrL^ neurons are not, during the retrieval of morphine withdrawal memory, suggesting that there may be a discontinuous retrieval of memory signals along a neural circuit. Another present finding that abnormal dopamine D1 receptor-ERK2 pathway plays a crucial role in context-induced increase in the expression of Arc and context-induced retrieval of morphine withdrawal memory may be of translational significance for prevent retrieval of morphine withdrawal memory.

However, the present study only reveals some diverse molecular pathways in BLA^-NAc^ neurons and BLA^-PrL^ neurons during context-induced retrieval of morphine withdrawal memory and we do not utilize single-cell RNA sequencing (scRNA-seq) technology to reveal more altered molecules for the model. Moving forward, studies are needed to utilize scRNA-seq to identify more molecular pathways involved in context-induced retrieval of morphine withdrawal memory in these two different projection neurons of the BLA.

Another limitation of the present study is the exclusive reliance on naloxone-induced measures of opioid withdrawal, which does not reflect the spontaneous opioid withdrawal that human patients would be experiencing. Opioid withdrawal can be expressed as both a spontaneous and precipitated syndrome [63]. The spontaneous opioid withdrawal is the more common clinical condition that human patients would be experiencing. The precipitated withdrawal is elicited by an opioid antagonist, such as naloxone, that is administered to an individual who is physically dependent on opioids. The naloxone-precipitated withdrawal is very similar to spontaneous withdrawal in dependent animals, except that the withdrawal is more dramatic and intense with the use of naloxone. In addition, in animal models of opioid withdrawal, the use of naloxone instead of spontaneous withdrawal from morphine allows pinpointing of the time of reduced opioid receptor activity [64]. Therefore, naloxone-precipitated withdrawal is widely used in animal withdrawal experiments. However, the conclusions would be more convincing if some cohorts of spontaneous withdrawal could be included in the experiments.

### Supplementary information

Supplementary Figures and legends

## Data Availability

All the data generated for this manuscript have been included in this published article.

## References

[1] Leshner AI (1997). Addiction is a brain disease, and it matters. Science.

[2] Koob GF, Volkow ND (2016). Neurobiology of addiction: a neurocircuitry analysis. Lancet Psychiatry.

[3] Robinson TE, Berridge KC (1993). The neural basis of drug craving: an incentive-sensitization theory of addiction. Brain Res Brain Res Rev.

[4] Koob GF (2021). Drug addiction: hyperkatifeia/negative reinforcement as a framework for medications development. Pharm Rev.

[5] Boning J (2009). Addiction memory as a specific, individually learned memory imprint. Pharmacopsychiatry.

[6] Lucas M, Frenois F, Vouillac C, Stinus L, Cador M, Le Moine C (2008). Reactivity and plasticity in the amygdala nuclei during opiate withdrawal conditioning: differential expression of c-fos and arc immediate early genes. Neuroscience.

[7] Frenois F, Stinus L, Di Blasi F, Cador M, Le Moine C (2005). A specific limbic circuit underlies opiate withdrawal memories. J Neurosci.

[8] He YY, Xue YX, Wang JS, Fang Q, Liu JF, Xue LF (2011). PKMzeta maintains drug reward and aversion memory in the basolateral amygdala and extinction memory in the infralimbic cortex. Neuropsychopharmacology.

[9] Hou YY, Lu B, Li M, Liu Y, Chen J, Chi ZQ (2009). Involvement of actin rearrangements within the amygdala and the dorsal hippocampus in aversive memories of drug withdrawal in acute morphine-dependent rats. J Neurosci.

[10] Li Y, Wang H, Qi K, Chen X, Li S, Sui N (2011). Orexins in the midline thalamus are involved in the expression of conditioned place aversion to morphine withdrawal. Physiol Behav.

[11] Dejean C, Sitko M, Girardeau P, Bennabi A, Caille S, Cador M (2017). Memories of opiate withdrawal emotional states correlate with specific gamma oscillations in the nucleus accumbens. Neuropsychopharmacology.

[12] Song J, Shao D, Guo X, Zhao Y, Cui D, Ma Q (2019). Crucial role of feedback signals from prelimbic cortex to basolateral amygdala in the retrieval of morphine withdrawal memory. Sci Adv.

[13] Zhao Y, Zhang J, Yang H, Cui D, Song J, Ma Q (2017). Memory retrieval in addiction: a role for miR-105-mediated regulation of D1 receptors in mPFC neurons projecting to the basolateral amygdala. BMC Biol.

[14] Zhu Y, Wienecke CF, Nachtrab G, Chen X (2016). A thalamic input to the nucleus accumbens mediates opiate dependence. Nature.

[15] Sheng H, Lei C, Yuan Y, Fu Y, Cui D, Yang L (2023). Nucleus accumbens circuit disinhibits lateral hypothalamus glutamatergic neurons contributing to morphine withdrawal memory in male mice. Nat Commun.

[16] Zhou K, Xu H, Lu S, Jiang S, Hou G, Deng X (2022). Reward and aversion processing by input-defined parallel nucleus accumbens circuits in mice. Nat Commun.

[17] Plath N, Ohana O, Dammermann B, Errington ML, Schmitz D, Gross C (2006). Arc/Arg3.1 is essential for the consolidation of synaptic plasticity and memories. Neuron.

[18] Chen M, Shao D, Fu Y, Ma Q, Chen M, Cui D (2019). Key determinants for morphine withdrawal conditioned context-induced increase in Arc expression in anterior cingulate cortex and withdrawal memory retrieval. Exp Neurol.

[19] Li M, Hou YY, Lu B, Chen J, Chi ZQ, Liu JG (2009). Expression pattern of neural synaptic plasticity marker-Arc in different brain regions induced by conditioned drug withdrawal from acute morphine-dependent rats. Acta Pharm Sin.

[20] Shen CJ, Zheng D, Li KX, Yang JM, Pan HQ, Yu XD (2019). Cannabinoid CB(1) receptors in the amygdalar cholecystokinin glutamatergic afferents to nucleus accumbens modulate depressive-like behavior. Nat Med.

[21] Butler RK, Sharko AC, Oliver EM, Brito-Vargas P, Kaigler KF, Fadel JR (2011). Activation of phenotypically-distinct neuronal subpopulations of the rat amygdala following exposure to predator odor. Neuroscience.

[22] Curran T, Morgan JI (1995). Fos: an immediate-early transcription factor in neurons. J Neurobiol.

[23] Impey S, Obrietan K, Storm DR (1999). Making new connections: role of ERK/MAP kinase signaling in neuronal plasticity. Neuron.

[24] Chotiner JK, Nielson J, Farris S, Lewandowski G, Huang F, Banos K (2010). Assessment of the role of MAP kinase in mediating activity-dependent transcriptional activation of the immediate early gene Arc/Arg3.1 in the dentate gyrus in vivo. Learn Mem.

[25] Waltereit R, Dammermann B, Wulff P, Scafidi J, Staubli U, Kauselmann G (2001). Arg3.1/Arc mRNA induction by Ca2+ and cAMP requires protein kinase A and mitogen-activated protein kinase/extracellular regulated kinase activation. J Neurosci.

[26] Ying SW, Futter M, Rosenblum K, Webber MJ, Hunt SP, Bliss TV (2002). Brain-derived neurotrophic factor induces long-term potentiation in intact adult hippocampus: requirement for ERK activation coupled to CREB and upregulation of Arc synthesis. J Neurosci.

[27] Girault JA, Valjent E, Caboche J, Herve D (2007). ERK2: a logical AND gate critical for drug-induced plasticity?. Curr Opin Pharm.

[28] Sweatt JD (2001). The neuronal MAP kinase cascade: a biochemical signal integration system subserving synaptic plasticity and memory. J Neurochem.

[29] Lv XF, Sun LL, Cui CL, Han JS. NAc shell Arc/Arg3.1 protein mediates reconsolidation of morphine CPP by increased GluR1 cell surface expression: activation of ERK-coupled CREB is required. Int J Neuropsychopharmacol. 2015;18:pyv030. 10.1093/ijnp/pyv030 PMC4576513 25746394

[30] Nikolaienko O, Eriksen MS, Patil S, Bito H, Bramham CR (2017). Stimulus-evoked ERK-dependent phosphorylation of activity-regulated cytoskeleton-associated protein (Arc) regulates its neuronal subcellular localization. Neuroscience.

[31] Borgkvist A, Valjent E, Santini E, Herve D, Girault JA, Fisone G (2008). Delayed, context- and dopamine D1 receptor-dependent activation of ERK in morphine-sensitized mice. Neuropharmacology.

[32] Gangarossa G, Di Benedetto M, O’Sullivan GJ, Dunleavy M, Alcacer C, Bonito-Oliva A (2011). Convulsant doses of a dopamine D1 receptor agonist result in Erk-dependent increases in Zif268 and Arc/Arg3.1 expression in mouse dentate gyrus. PLoS One.

[33] Nagai T, Yoshimoto J, Kannon T, Kuroda K, Kaibuchi K (2016). Phosphorylation signals in striatal medium spiny neurons. Trends Pharm Sci.

[34] Li Z, Luan W, Chen Y, Chen M, Dong Y, Lai B (2011). Chronic morphine treatment switches the effect of dopamine on excitatory synaptic transmission from inhibition to excitation in pyramidal cells of the basolateral amygdala. J Neurosci.

[35] de Oliveira AR, Reimer AE, de Macedo CE, de Carvalho MC, Silva MA, Brandao ML (2011). Conditioned fear is modulated by D2 receptor pathway connecting the ventral tegmental area and basolateral amygdala. Neurobiol Learn Mem.

[36] Satoh T, Nakatsuka D, Watanabe Y, Nagata I, Kikuchi H, Namura S (2000). Neuroprotection by MAPK/ERK kinase inhibition with U0126 against oxidative stress in a mouse neuronal cell line and rat primary cultured cortical neurons. Neurosci Lett.

[37] Sah P, Faber ES, Lopez De Armentia M, Power J (2003). The amygdaloid complex: anatomy and physiology. Physiol Rev.

[38] Burgos-Robles A, Kimchi EY, Izadmehr EM, Porzenheim MJ, Ramos-Guasp WA, Nieh EH (2017). Amygdala inputs to prefrontal cortex guide behavior amid conflicting cues of reward and punishment. Nat Neurosci.

[39] Bradizza CM, Stasiewicz PR, Paas ND (2006). Relapse to alcohol and drug use among individuals diagnosed with co-occurring mental health and substance use disorders: a review. Clin Psychol Rev.

[40] Beyeler A, Namburi P, Glober GF, Simonnet C, Calhoon GG, Conyers GF (2016). Divergent routing of positive and negative information from the Amygdala during memory retrieval. Neuron.

[41] Namburi P, Beyeler A, Yorozu S, Calhoon GG, Halbert SA, Wichmann R (2015). A circuit mechanism for differentiating positive and negative associations. Nature.

[42] Stefanik MT, Kalivas PW (2013). Optogenetic dissection of basolateral amygdala projections during cue-induced reinstatement of cocaine seeking. Front Behav Neurosci.

[43] Stuber GD, Sparta DR, Stamatakis AM, van Leeuwen WA, Hardjoprajitno JE, Cho S (2011). Excitatory transmission from the amygdala to nucleus accumbens facilitates reward seeking. Nature.

[44] Ramirez F, Moscarello JM, LeDoux JE, Sears RM (2015). Active avoidance requires a serial basal amygdala to nucleus accumbens shell circuit. J Neurosci.

[45] Zhang X, Guan W, Yang T, Furlan A, Xiao X, Yu K (2021). Genetically identified amygdala-striatal circuits for valence-specific behaviors. Nat Neurosci.

[46] Kim J, Pignatelli M, Xu S, Itohara S, Tonegawa S (2016). Antagonistic negative and positive neurons of the basolateral amygdala. Nat Neurosci.

[47] Tzingounis AV, Nicoll RA (2006). Arc/Arg3.1: linking gene expression to synaptic plasticity and memory. Neuron.

[48] Alaghband Y, O’Dell SJ, Azarnia S, Khalaj AJ, Guzowski JF, Marshall JF (2014). Retrieval-induced NMDA receptor-dependent Arc expression in two models of cocaine-cue memory. Neurobiol Learn Mem.

[49] Nakayama D, Iwata H, Teshirogi C, Ikegaya Y, Matsuki N, Nomura H (2015). Long-delayed expression of the immediate early gene Arc/Arg3.1 refines neuronal circuits to perpetuate fear memory. J Neurosci.

[50] Rosenegger D, Lukowiak K (2010). The participation of NMDA receptors, PKC, and MAPK in the formation of memory following operant conditioning in Lymnaea. Mol Brain.

[51] Atkins CM, Selcher JC, Petraitis JJ, Trzaskos JM, Sweatt JD (1998). The MAPK cascade is required for mammalian associative learning. Nat Neurosci.

[52] English JD, Sweatt JD (1996). Activation of p42 mitogen-activated protein kinase in hippocampal long term potentiation. J Biol Chem.

[53] Fiore RS, Bayer VE, Pelech SL, Posada J, Cooper JA, Baraban JM (1993). p42 mitogen-activated protein kinase in brain: prominent localization in neuronal cell bodies and dendrites. Neuroscience.

[54] Giovannini MG (2006). The role of the extracellular signal-regulated kinase pathway in memory encoding. Rev Neurosci.

[55] Yoon S, Seger R (2006). The extracellular signal-regulated kinase: multiple substrates regulate diverse cellular functions. Growth Factors.

[56] Peng S, Zhang Y, Zhang J, Wang H, Ren B (2010). ERK in learning and memory: a review of recent research. Int J Mol Sci.

[57] Di Cristo G, Berardi N, Cancedda L, Pizzorusso T, Putignano E (2001). Requirement of ERK activation for visual cortical plasticity. Science.

[58] English JD, Sweatt JD (1997). A requirement for the mitogen-activated protein kinase cascade in hippocampal long term potentiation. J Biol Chem.

[59] Boulton TG, Nye SH, Robbins DJ, Ip NY, Radziejewska E, Morgenbesser SD (1991). ERKs: a family of protein-serine/threonine kinases that are activated and tyrosine phosphorylated in response to insulin and NGF. Cell.

[60] Janak PH, Tye KM (2015). From circuits to behaviour in the amygdala. Nature.

[61] Tucci S, Rada P, Hernandez L (1998). Role of glutamate in the amygdala and lateral hypothalamus in conditioned taste aversion. Brain Res.

[62] Osorio-Gomez D, Guzman-Ramos K, Bermudez-Rattoni F (2016). Differential involvement of glutamatergic and catecholaminergic activity within the amygdala during taste aversion retrieval on memory expression and updating. Behav Brain Res.

[63] Dunn KE, Bird HE, Bergeria CL, Ware OD, Strain EC, Huhn AS (2023). Operational definition of precipitated opioid withdrawal. Front Psychiatry.

[64] Bechara A, Nader K, van der Kooy D (1995). Neurobiology of withdrawal motivation: evidence for two separate aversive effects produced in morphine-naive versus morphine-dependent rats by both naloxone and spontaneous withdrawal. Behav Neurosci.

