# Supplementary material for: Different projection neurons of basolateral amygdala participate in the retrieval of morphine withdrawal memory with diverse molecular pathways
Source: Mol Psychiatry. 2023 Dec 26;29(3):793–808. doi: 10.1038/s41380-023-02371-x (PMC11153146; doi:10.1038/s41380-023-02371-x)
Supplement: Supplementary file 1 — Supplementary Figures and legends [file 41380_2023_2371_MOESM1_ESM.docx]

**Different projection neurons of basolateral amygdala participate in the retrieval of morphine withdrawal memory with diverse molecular pathways**

***Supplementary Material***

Fig. S1. BLA^-PrL^ and BLA^-NAc^ neurons are glutamatergic neurons. Related to Figure 1.

Fig. S2. Anatomical location of FG injection site **of the mice** **used in the experiment to test c-Fos and Arc expression.** Related to Figure 1.

Fig. S3. Anatomical location of cre-mCherry and hM4Di-EGFP injection site **of the mice** **used in the experiment to chemogenetic inhibition of BLA^-PrL^ or BLA-^NAc^ neurons.** Related to Figure 2.

**Fig. S4. Anatomical location of FG injection site and the CPA scores of the mice used in the experiment to test Arc and D1 mRNA levels.** Related to Figure 3 and 5.

**Fig. S5. Anatomical location of FG injection site, the CPA scores and HTFR ratio of the mice** **used in the experiment to test pERK expression.** Related to Figure 4.

Fig. S6. Anatomical location of cre-mCherry, MIR30-shERK2-EGFP, MIR30-shERK1-EGFP injection site, and **the CPA scores of the mice** **used in the experiment to test the relationship of Arc, pERK and D1 receptors.** Related to Figure 5.

Fig. S7. Anatomical location of SCH23390/Sulpiride injection site, and **the CPA scores of the mice** **used in the experiment to test the relationship of Arc, pERK and D1 receptors.** Related to Figure 5.

Fig. S8. Anatomical location of microsphere injection site and **the CPA scores of the mice** **used in electrophysiological experiments.** Related to Figure 6.

**
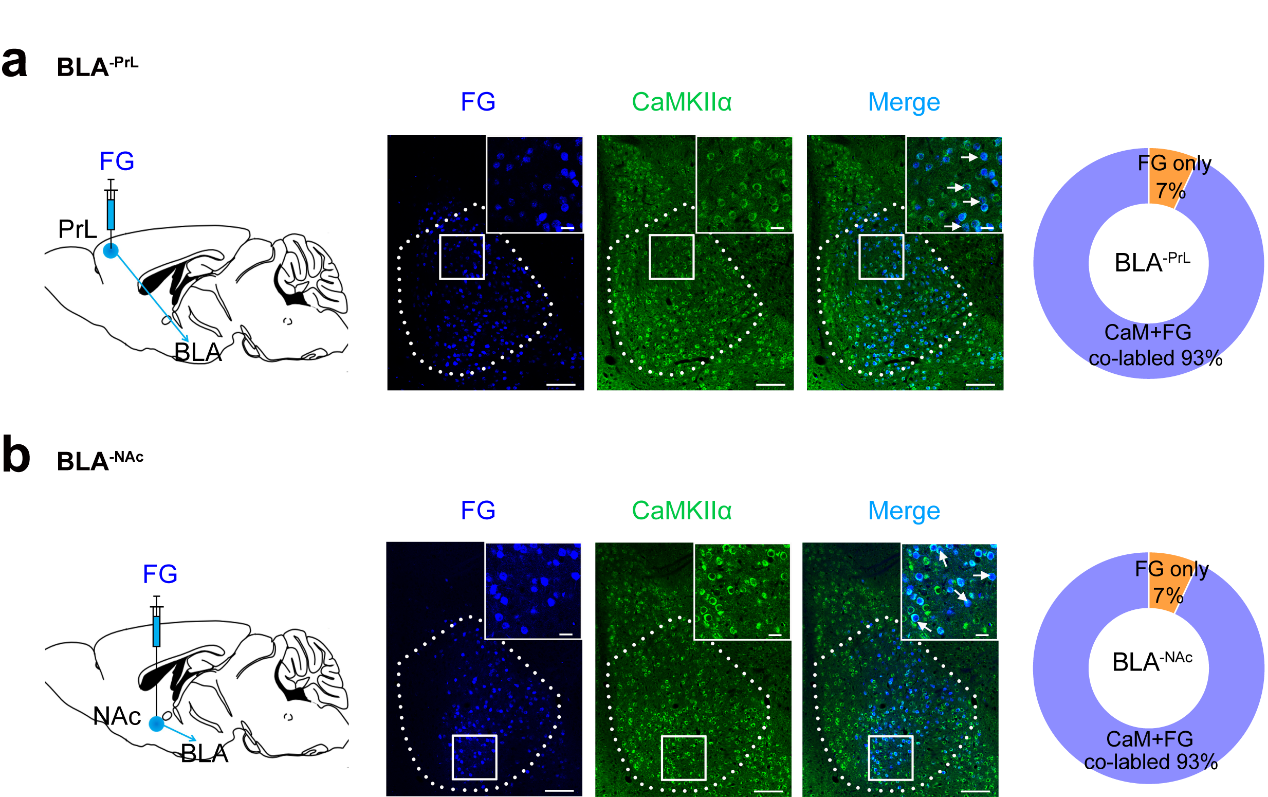
**

Fig. S1. BLA^-PrL^ and BLA^-NAc^ neurons are glutamatergic neurons. Related to Figure 1.

(**a**) Left: diagram of the injection of fluorogold (FG) into the PrL. Middle: left, FG-labeling neurons in the BLA; middle, CaMKIIα-positive neurons in the BLA; right, co-labeling neurons of CaMKIIα and FG in the BLA. Scale bar, 100 μm. Magnified image shows the boxed area. Scale bar, 20 μm. Right: quantification of co-retrieval of CaMKIIα and FG relative to FG in the BLA. n = 6 mice.

(**b**) Left: diagram of the injection of FG into the NAc. Middle: left, FG-labeling neurons in the BLA; middle, CaMKIIα-positive neurons in the BLA; right, co-labeling neurons of CaMKIIα and FG in the BLA. Scale bar, 100 μm. Magnified image shows the boxed area. Scale bar, 20 μm. Right: quantification of co-retrieval of CaMKIIα and FG relative to FG in the BLA. n = 5 mice. Means ± SEMs.


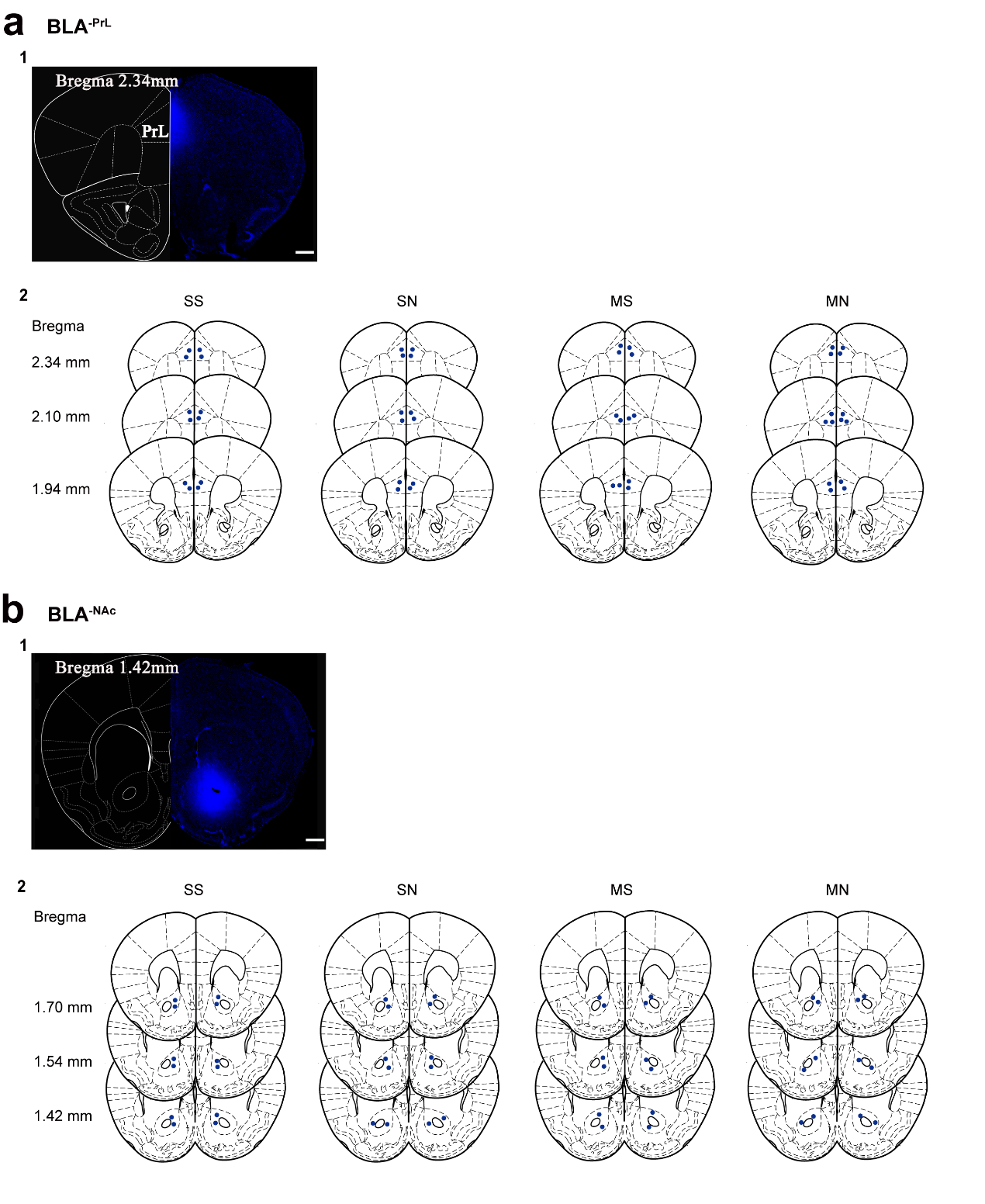


Fig. S2. Anatomical location of FG injection site **of the mice** **used in the experiment to test c-Fos and Arc expression.** Related to Figure 1.

(**a1, 2**) Anatomical location of FG (blue) injection site in PrL. Scale bar, 500 μm.

(**b1, 2**) Anatomical location of FG (blue) injection site in NAc. Scale bar, 500 μm.


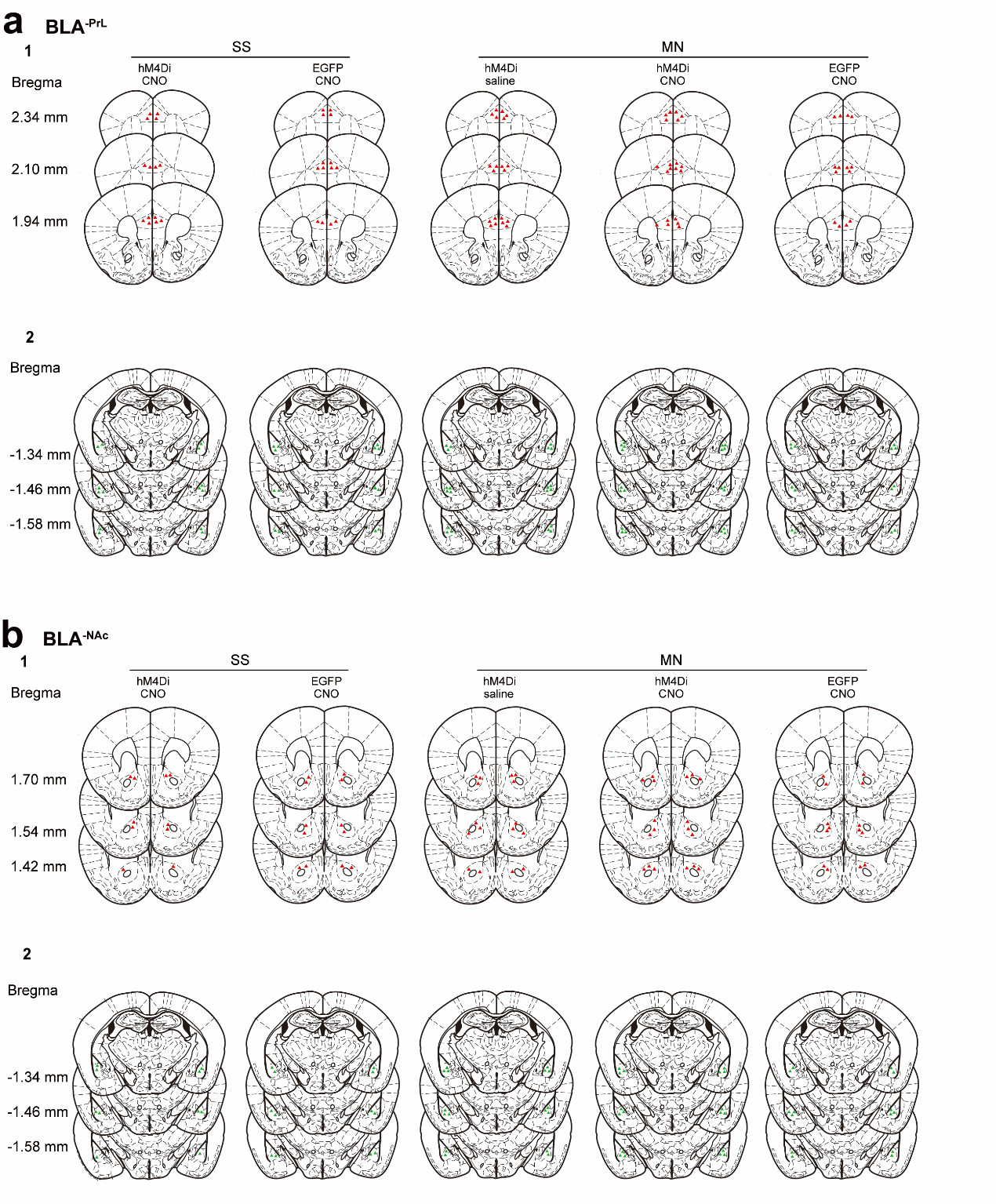


Fig. S3. Anatomical location of cre-mCherry and hM4Di-EGFP injection site **of the mice** **used in the experiment to chemogenetic inhibition of** BLA^-PrL^ or BLA^-NAc^ neurons**.** Related to Figure 2.

(**a1**) Anatomical location of cre-mCherry (red) injection site in PrL in the experiment to chemogenetic inhibition of BLA^-PrL^ neurons. (**a2**) Anatomical location of hM4Di-EGFP (green) injection site in BLA in the experiment to chemogenetic inhibition of BLA^-PrL^ neurons.

(**b1**) Anatomical location of cre-mCherry (red) injection site in NAc in the experiment to chemogenetic inhibition of BLA^-NAc^ neurons. (**b2**) Anatomical location of hM4Di-EGFP (green) injection site in BLA in the experiment to chemogenetic inhibition of BLA^-NAc^ neurons.


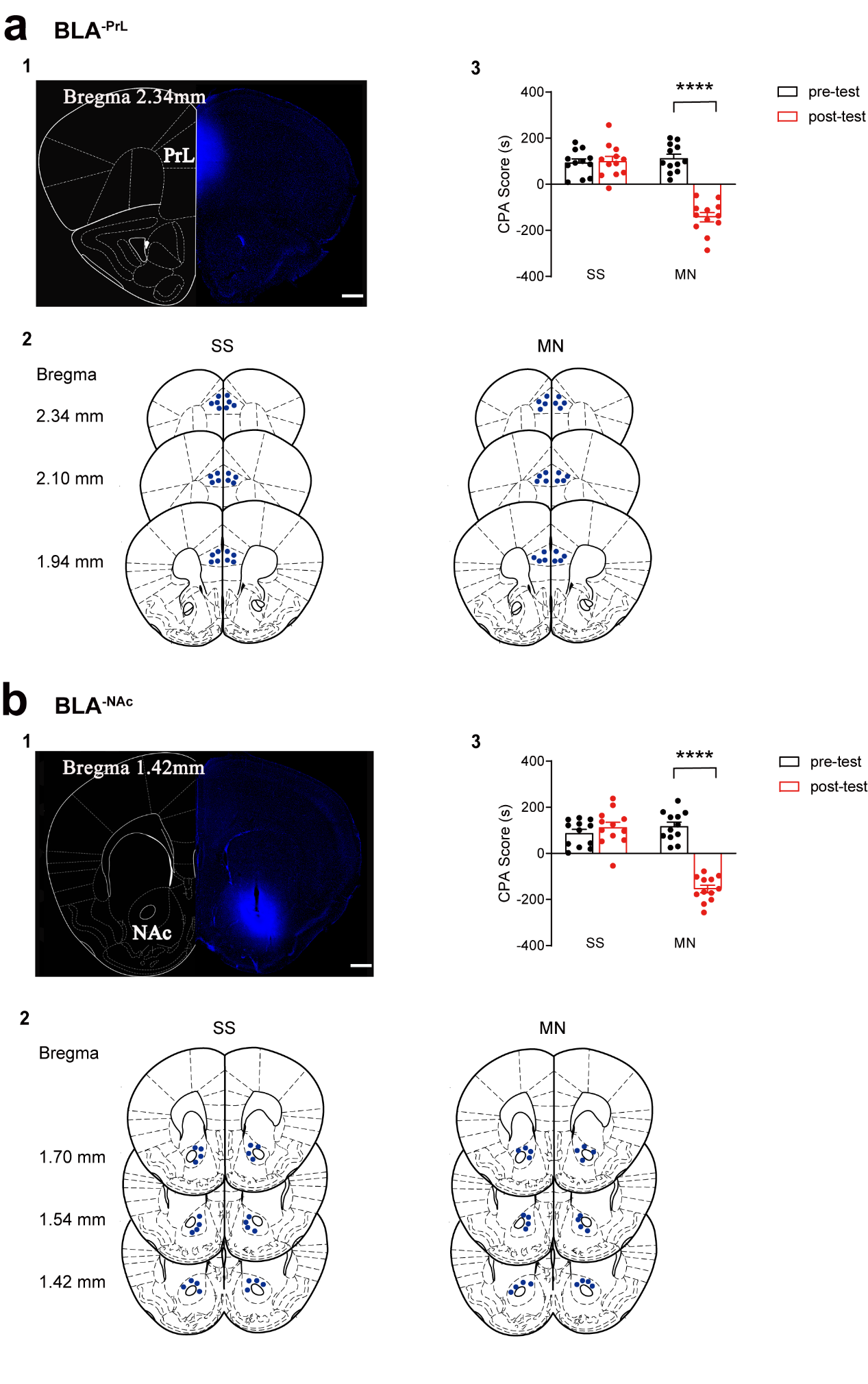


Fig. S4. Anatomical location of FG injection site and **the CPA scores of the mice** **used in the experiment to test Arc and D1 mRNA levels.** Related to Figure 3.

(**a1, 2**) Anatomical location of FG (blue) injection site in the PrL. Scale bar, 500 μm. (**a3**) The CPA scores of the mice that were used to test Arc and D1 mRNA levels of BLA^-PrL^ neurons. Average CPA scores in SS (n = 12) and MN (n = 12) groups. Two-way ANOVA, **** P < 0.0001.

(**b1, 2**) Anatomical location of FG (blue) injection site in the NAc. Scale bar, 500 μm. (**b3**) The CPA scores of the mice that were used to test Arc and D1 mRNA levels of BLA^-PrL^ neurons BLA^-NAc^ neurons. Average CPA scores in SS (n = 12) and MN (n = 12) groups. Two-way ANOVA, **** P < 0.0001.

**
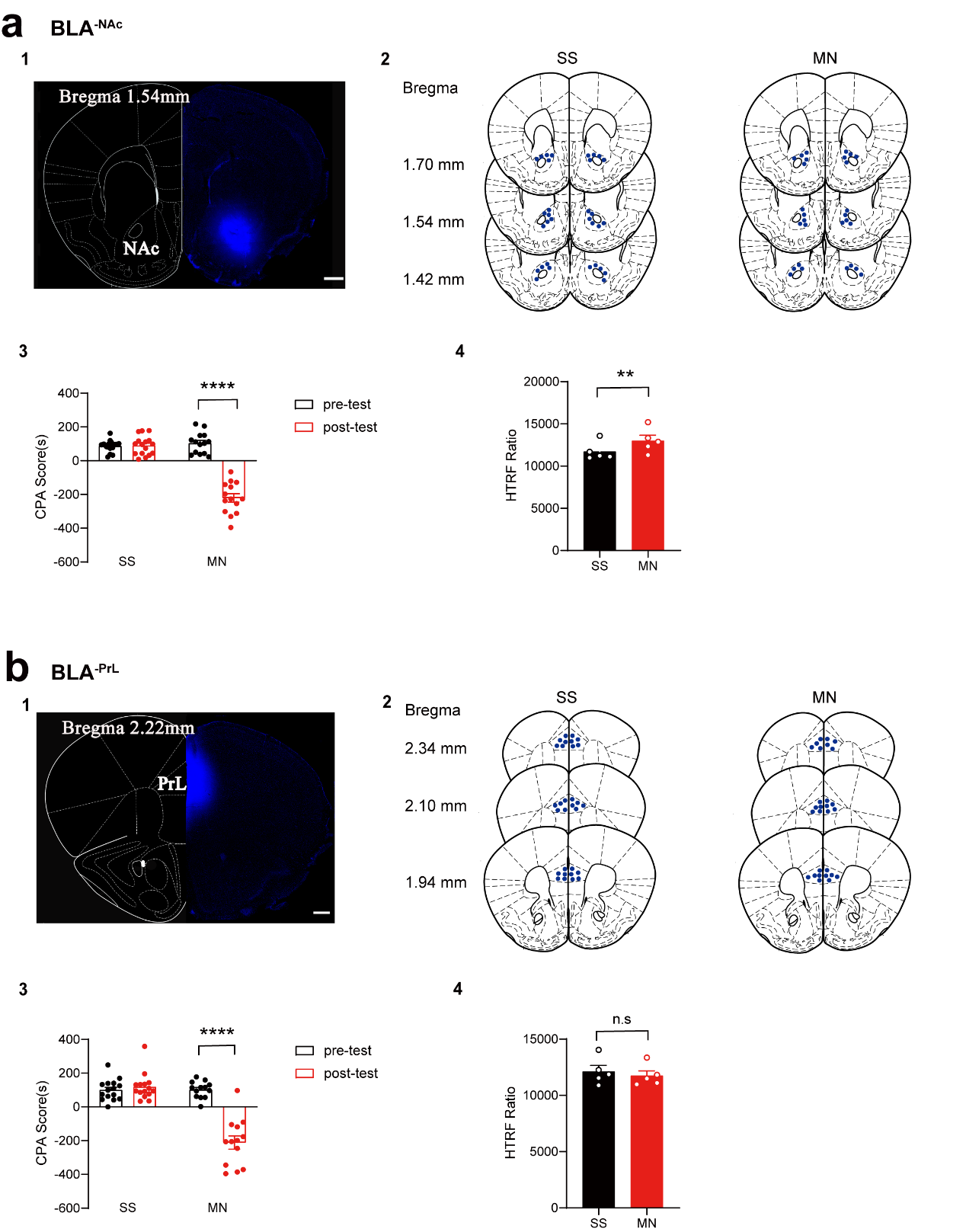
**

Fig. S5. Anatomical location of FG injection site, **the CPA scores and HTFR ratio of the mice** **used in the experiment to test pERK expression.** Related to Figure 4.

(**a1, 2**) Anatomical location of FG (blue) injection site in the NAc. Scale bar, 500 μm. (**a3**) The CPA scores of the mice that were used to test pERK expression using HFTR of BLA^-NAc^ neurons**.** Average CPA scores in SS (n = 16) and MN (n = 14) groups. Two-way ANOVA, **** P < 0.0001. (**a4**) HTFR ratio of pERK in BLA^-NAc^ neurons (n = 5 in each group). Paired t test, ** P < 0.01.

(**b1, 2**) Anatomical location of FG (blue) injection site in the PrL. Scale bar, 500 μm. (**b3**) The CPA scores of the mice that were used to test pERK expression using HFTR of BLA^-PrL^ neurons**.** Average CPA scores in SS (n = 15) and MN (n = 13) groups. Two-way ANOVA, **** P < 0.0001. (**b4**) HTFR ratio of pERK in BLA^-PrL^ neurons (n = 5 in each group). Paired t test, P > 0.05.

**
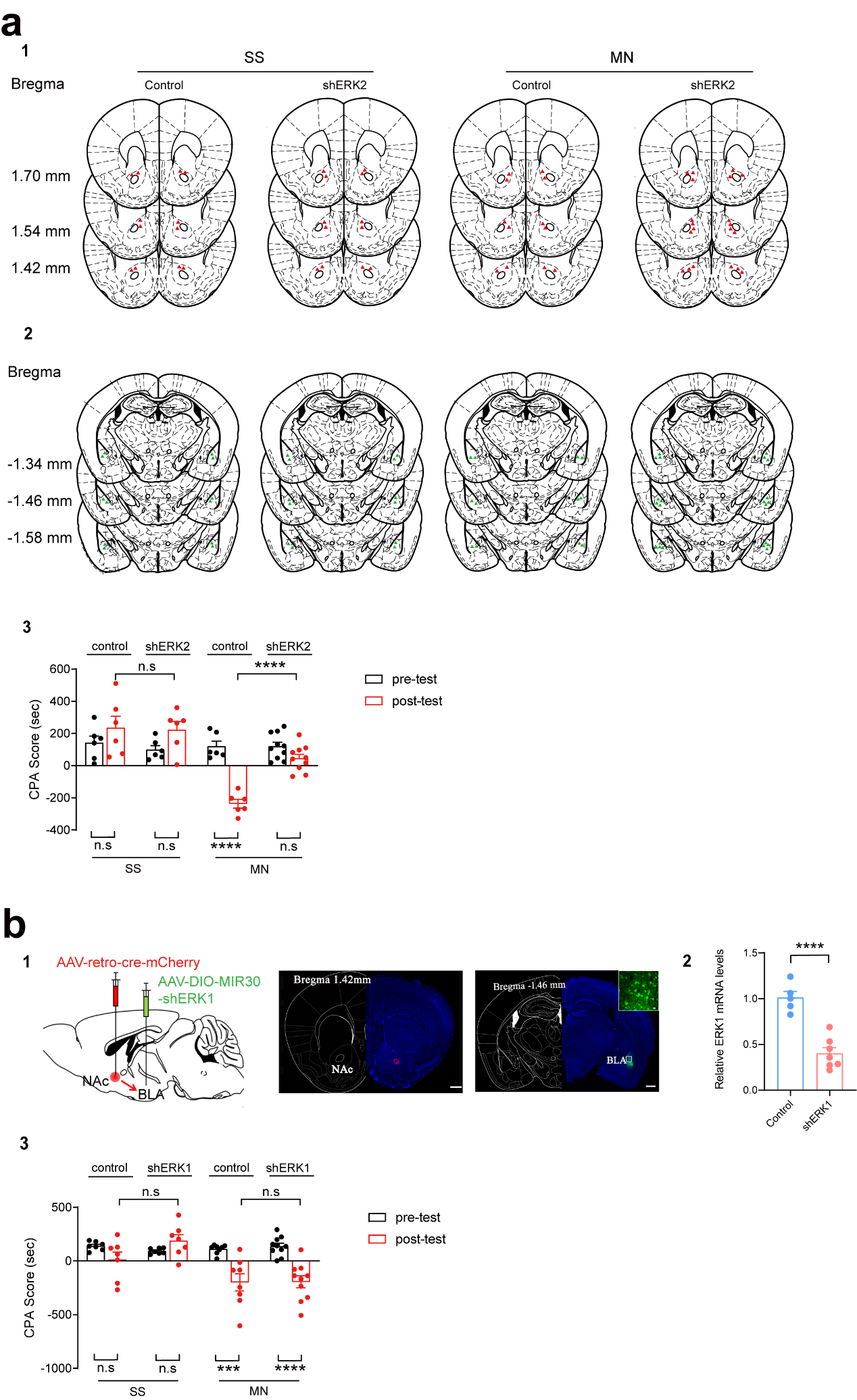
**

Fig. S6. Anatomical location of cre-mCherry, MIR30-shERK2-EGFP, MIR30-shERK1-EGFP injection site, and **the CPA scores of the mice** **used in the experiment to test the relationship of Arc, pERK and D1 receptors.** Related to Figure 5.

(**a1**) Anatomical location of cre-mCherry (red) injection site in NAc. (**a2**) Anatomical location of MIR30-shERK2-EGFP (green) injection site in BLA. (**a3**) The CPA scores of the mice used in shERK2 experiment. Average CPA scores in SS (control) (n = 6), SS (shERK2) (n = 6), MN (control) (n = 6) and MN (shERK2) (n = 10) groups. Two-way ANOVA, **** P < 0.0001.

(**b1**) Left: diagram of the injection of virus into the BLA and NAc. Middle: anatomical locations of the injection site of cre-mCherry (red) in the NAc. Right: the expression of MIR30-shERK1-EGFP (green) in the BLA. Scale bar, 500 μm. (**b2**) The averaged ERK1 mRNA expression level in BLA in the control and shERK1 groups by qRT-PCR. Unpaired t test, **** P < 0.0001. (**b3**) The CPA scores of the mice used in ERK1-shRNA experiment. Average CPA scores in SS (control) (n = 7), SS (shERK1) (n = 7), MN (control) (n = 8) and MN (shERK1) (n = 10) groups. Two-way ANOVA, *** P < 0.001.


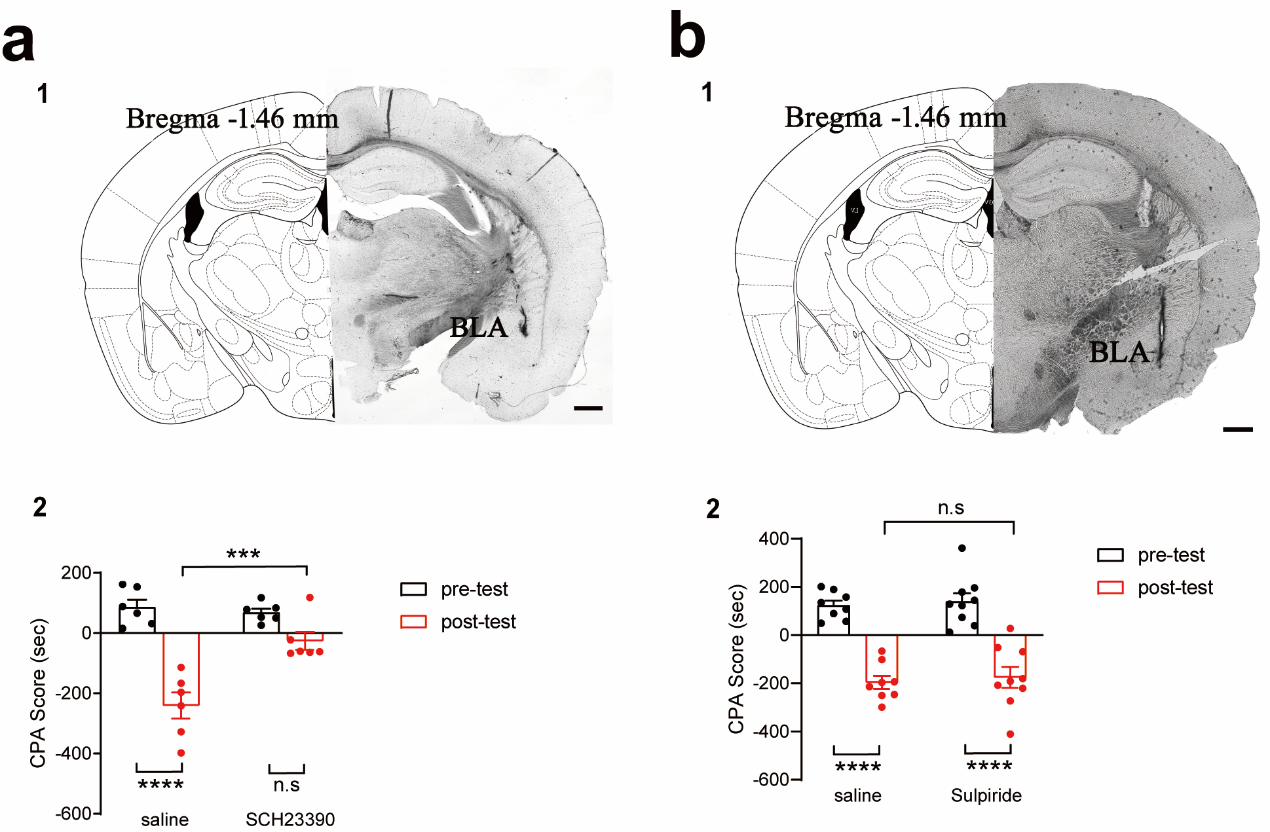


Fig. S7. Anatomical location of SCH23390/Sulpiride injection site, and **the CPA scores of the mice** **used in the experiment to test the relationship of Arc, pERK and D1 receptors.** Related to Figure 5.

(**a1**) Anatomical location of SCH23390 injection site in BLA. Scale bar, 500 μm. (**a2**) The CPA scores of the mice used in D1 inhibition experiment by SCH23390 application. Average CPA scores in saline (n = 6) and SCH23390 (n = 6) groups. Two-way ANOVA, *** P < 0.01, **** P < 0.0001.

(**b1**) Anatomical location of sulpiride injection site in BLA. Scale bar, 500 μm. (**b2**) The CPA scores of the mice used in D2 inhibition experiment by sulpiride application. Average CPA scores in saline (n = 8) and sulpiride (n = 9) groups. Two-way ANOVA, **** P < 0.0001.


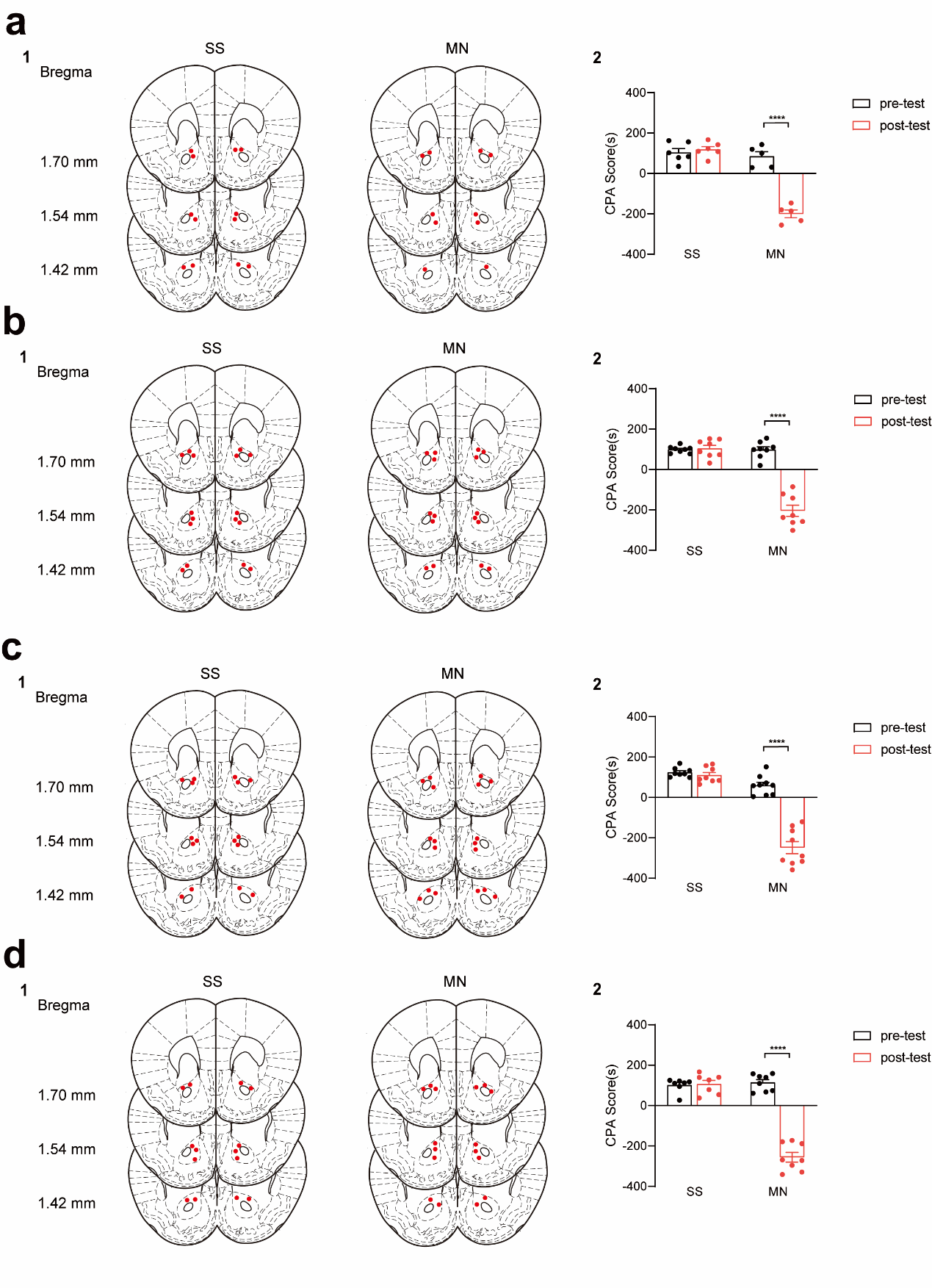


Fig. S8. Anatomical location of microsphere injection site and **the CPA scores of the mice** **used in electrophysiological experiments.** Related to Figure 6.

(**a1, 2**) Anatomical location of microsphere (red) injection site in the NAc and the CPA scores of the mice used in mEPSC recoding experiment of BLA^-NAc^ neurons. Average CPA scores in SS (n = 6) and MN (n = 5) groups. Two-way ANOVA, **** P < 0.0001. (**b1, 2**) Anatomical location of microsphere (red) injection site in the NAc and the CPA scores of the mice used in AMPA current experiment of BLA^-NAc^ neurons. Average CPA scores in SS (n = 8) and MN (n = 8) groups. Two-way ANOVA, **** P < 0.0001.

(**c1,2**) Anatomical location of microsphere (red) injection site in the NAc and the CPA scores of the mice used in AMPA current experiment （SKF38393/U0126）of BLA-NAc neurons. Average CPA scores in SS (n = 8) and MN (n = 9) groups. Two-way ANOVA, **** P < 0.0001.

(**d1, 2**) Anatomical location of microsphere (red) injection site in the NAc and the CPA scores of the mice used in NMDA current experiment of BLA^-NAc^ neurons. Average CPA scores in SS (n = 7) and MN (n = 8) groups. Two-way ANOVA, **** P < 0.0001.
